# Integrated analysis of single-cell and bulk RNA sequencing data reveals a pan-cancer stemness signature predicting immunotherapy response

**DOI:** 10.1186/s13073-022-01050-w

**Published:** 2022-04-29

**Authors:** Zhen Zhang, Zi-Xian Wang, Yan-Xing Chen, Hao-Xiang Wu, Ling Yin, Qi Zhao, Hui-Yan Luo, Zhao-Lei Zeng, Miao-Zhen Qiu, Rui-Hua Xu

**Affiliations:** 1 Department of Medical Oncology, Sun Yat-sen University Cancer Center, State Key Laboratory of Oncology in South China, Collaborative Innovation Center for Cancer Medicine, Sun Yat-sen University, Guangzhou, 510060 P. R. China; 2 Research Unit of Precision Diagnosis and Treatment for Gastrointestinal Cancer, Chinese Academy of Medical Sciences, Guangzhou, 510060 P. R. China; 3 Laboratory of Artificial Intelligence and Data Science, Sun Yat-sen University Cancer Center, Guangzhou, 510060 P. R. China

**Keywords:** Big data analysis, Single-cell sequencing, Immune checkpoint therapy, Stemness, Pan-cancer

## Abstract

**Background:**

Although immune checkpoint inhibitor (ICI) is regarded as a breakthrough in cancer therapy, only a limited fraction of patients benefit from it. Cancer stemness can be the potential culprit in ICI resistance, but direct clinical evidence is lacking.

**Methods:**

Publicly available scRNA-Seq datasets derived from ICI-treated patients were collected and analyzed to elucidate the association between cancer stemness and ICI response. A novel stemness signature (Stem.Sig) was developed and validated using large-scale pan-cancer data, including 34 scRNA-Seq datasets, The Cancer Genome Atlas (TCGA) pan-cancer cohort, and 10 ICI transcriptomic cohorts. The therapeutic value of Stem.Sig genes was further explored using 17 CRISPR datasets that screened potential immunotherapy targets.

**Results:**

Cancer stemness, as evaluated by CytoTRACE, was found to be significantly associated with ICI resistance in melanoma and basal cell carcinoma (both *P* < 0.001). Significantly negative association was found between Stem.Sig and anti-tumor immunity, while positive correlations were detected between Stem.Sig and intra-tumoral heterogenicity (ITH) / total mutational burden (TMB). Based on this signature, machine learning model predicted ICI response with an AUC of 0.71 in both validation and testing set. Remarkably, compared with previous well-established signatures, Stem.Sig achieved better predictive performance across multiple cancers. Moreover, we generated a gene list ranked by the average effect of each gene to enhance tumor immune response after genetic knockout across different CRISPR datasets. Then we matched Stem.Sig to this gene list and found Stem.Sig significantly enriched 3% top-ranked genes from the list (*P* = 0.03), including EMC3, BECN1, VPS35, PCBP2, VPS29, PSMF1, GCLC, KXD1, SPRR1B, PTMA, YBX1, CYP27B1, NACA, PPP1CA, TCEB2, PIGC, NR0B2, PEX13, SERF2, and ZBTB43, which were potential therapeutic targets.

**Conclusions:**

We revealed a robust link between cancer stemness and immunotherapy resistance and developed a promising signature, Stem.Sig, which showed increased performance in comparison to other signatures regarding ICI response prediction. This signature could serve as a competitive tool for patient selection of immunotherapy. Meanwhile, our study potentially paves the way for overcoming immune resistance by targeting stemness-associated genes.

**Supplementary Information:**

The online version contains supplementary material available at 10.1186/s13073-022-01050-w.

## Background

Immune checkpoint inhibitor (ICI) has ushered in a new era of cancer treatment and provided unprecedented clinical benefits for patients [1]. However, only a relatively small proportion of patients respond to it [2], which highlights the necessity of biomarker research for optimizing patient selection and combination strategies to tackle immune resistance.

Traditional biomarker research mostly focused on the analysis of whole exome sequencing (WES) or RNA sequencing (RNA-Seq) from intact tumor tissue (bulk data) [3–8], which only reflects the average genetic profile across a large population of different cells. Pre-existing ICI biomarkers derived from these studies showed limited predictive values. The development of single-cell RNA sequencing (scRNA-Seq) enables us to dissect gene expression at single-cell resolution and identify novel biomarkers with better performance [9].

Cancer stem cells (CSCs) are self-renewal cells that promote tumor initiation, progression, and metastasis [10]. Mounting evidences revealed a prominent association between stemness and cancer immune evasion and resistance [11]. A previous study demonstrated that high stemness correlates with immune cell exclusion across 21 solid cancer types [12], but direct clinical evidence validating the negative association between stemness and ICI outcomes is lacking. With the help of a powerful computational framework (CytoTRACE) developed by Gulati et al., we can accurately characterize cancer stemness and identify stemness-correlated genes at the resolution of single-cell level to better investigate the impacts of stemness on ICI [13].

In this study, we revealed and verified the negative association between cancer stemness and ICI outcomes in two scRNA-Seq ICI cohorts [14, 15]. Thereafter a stemness signature (Stem.Sig) was developed through an integrative analysis of 34 scRNA-Seq datasets, which consisted of 345 patients and 663,760 cells across 17 cancer types [14–43]. The predictive value of Stem.Sig was further explored and validated through a comprehensive analysis of pan-cancer transcriptomic data (10,154 patients; 30 cancer types) [44], 17 CRISPR datasets (4 cancer types) [45–51], and 10 independent ICI cohorts (921 patients; 5 cancer types) [52–61]. Our findings uncovered the potential of Stem.Sig for predicting ICI outcomes more accurately than previously recognized signatures across multiple cancer types.

## Methods

### scRNA-Seq ICI cohorts

To investigate the relationship between cancer cell stemness and immunotherapy efficacy, a melanoma cohort with both ICI response and scRNA-Seq data was analyzed [14, 15]. Another independent scRNA-seq ICI cohort of basal cell carcinoma was used to validate the results. Data of these two cohorts was accessed through GEO accession number: GSE115978 [14] and GSE123813 [15], respectively (Additional file 1: Table S1).

### Pan-cancer scRNA-Seq datasets

For the development of stemness signature (Stem.Sig), 34 scRNA-Seq datasets with both malignant and stromal/immune cells data were collected from the TISCH portal (http://tisch.comp-genomics.org/) [62], which consist of 345 patients and 663,760 cells (Additional file 1: Table S2). These datasets cover 17 cancer types, including basal cell carcinoma (BCC), breast cancer (BRCA), cholangiocarcinoma (CHOL), colorectal cancer (CRC), glioma, head and neck cancer (HNSC), liver hepatocellular carcinoma (LIHC), medulloblastoma (MB), Merkel cell carcinoma (MCC), multiple myeloma (MM), neuroendocrine tumor (NET), non-small cell lung cancer (NSCLC), ovarian serous cystadenocarcinoma (OV), pancreatic adenocarcinoma (PAAD), skin cutaneous melanoma (SKCM), stomach adenocarcinoma (STAD), and uveal melanoma (UVM) [14–43].

### Pan-cancer transcriptomic data

Transcriptomic data of The Cancer Genome Atlas (TCGA) Pan-cancer cohort was downloaded from the UCSC Xena data portal (https://xenabrowser.net) [44] to explore the potential links between Stem.Sig and immune suppression across 30 different cancer types. Three cancer types were excluded from our analysis, including diffuse large B cell lymphoma (DLBC), acute myeloid leukemia (LAML), and thymoma (THYM), as they mainly consist of immune cells [63]. Total mutation burden (TMB) was retrieved from cBioPortal (https://www.cbioportal.org) [64, 65] and intratumor heterogeneity (ITH) data was from Thorsson et al. [66], which were used for analyzing the correlation between Stem.Sig and TMB or ITH.

### ICI RNA-Seq cohorts

To validate the predictive value of Stem.Sig, we systemically collected transcriptomic data and clinical information of pretreatment samples from 10 ICI RNA-Seq cohorts, including 5 SKCM cohorts (Hugo 2016 [59], Liu 2019 [54], Gide 2019 [55], Riaz 2017 [56], Van Allen 2015 [60]), 2 urothelial carcinoma (UC) cohorts (Mariathasan 2018 [53], Synder 2017 [58]), 1 glioblastoma multiform (GBM) cohort (Zhao 2019 [57]), 1 gastric cancer (GC) cohort (Kim 2018 [61]) and 1 renal cell carcinoma (RCC) cohort (Braun 2020 [52]). Anti-PD-1 therapy, anti-PD-L1 therapy, anti-CTLA4 therapy, and anti-PD-(L)1 plus anti-CTLA-4 combination therapy were employed in 6, 2, 1, and 1 cohort, respectively. Cohort Hugo 2016 [59] comprises 27 pre-treated tumor samples from 26 patients, while cohort Zhao 2019 [57] consists of 34 pre-treated tumor samples from 17 patients. In these two cohorts, we randomly selected a single tumor sample for each corresponding patient. The details of these cohorts are summarized in Additional file 1: Table S3.

### CRISPR screening data

To explore potential therapeutic targets of Stem.Sig genes, we collected data from 7 published CRISPR/Cas9 screening studies that assessed the individual effect of each gene knockout on tumor immunity, including Freeman 2019 [47], Kearney 2018 [46], Manguso 2017 [51], Pan 2018 [50], Patel 2017 [49], Vredevoogd 2019 [48], and Lawson 2020 [45]. The first six CRISPR studies have been previously curated by Fu et al. [8] In addition to Fu et al., we further collected another CRISPR cohort from Lawson et al. [45]. According to model cell lines and treatment conditions applied, these seven CRISPR studies were divided into 17 datasets (Additional file 1: Table S4). The CRISPR analysis covers melanoma, breast cancer, colon cancer, and renal cancer cell lines. We utilized these data to identify genes that are more likely to modulate lymphocyte-mediated cancer killing and influence immunotherapy response across different datasets.

The process of CRISPR screens is to perform genome-wide CRISPR-Cas9 knockout across various cancer cell lines that were co-cultured with/without cytotoxic lymphocytes (CTLs) in vitro or implanted into immune-deficient mice/immune-competent mice in vivo. Then RNA sequencing is used to estimate the abundance of sgRNA targeting the corresponding gene. To measure the effect of gene knockout on cancer fitness under the pressure of CTLs or anti-tumor immunity, log-fold changes of sgRNA reads are calculated for paired screens of cell lines (with CTLs vs. without CTLs; immune-deficient mice vs. immune-competent mice) [45]. Normalized *z* scores were called from the log-fold changes in order to remove batch effects and compare genes among CRISPR datasets from different studies. The lower *z* scores indicate better immune response after gene knockout. We also ranked genes based on the average *z* scores across 17 datasets. Top-ranked genes with lower *z* scores were characterized as immune resistant.

### scRNA-Seq data analysis

Previous studies revealed a global reduction of chromatin accessibility during lineage commitment [13]. Since chromatin accessibility could be quantitatively reflected by single-cell gene counts, Gulati et al. had discovered a prominent association between single-cell gene counts and differential status of the corresponding cell [13]. Higher single-cell gene counts correlate with less cellular differentiation (higher stemness). The CytoTRACE algorithm is developed by Gulati et al. to capture, smooth, and calculate the expression level of genes that are most highly correlated with single-cell gene counts with scRNA-Seq data. When the calculation of the CytoTRACE algorithm is finished, each single cell will get a score that represents its stemness within the given dataset. CytoTRACE is a robust computational framework for differentiation states prediction via scRNA-seq data, which was validated in large-scale datasets and outperformed pre-existing computational techniques of stemness [13]. R package *CytoTRACE* v0.3.3 was applied to calculate the CytoTRACE scores for malignant cells. CytoTRACE scores range from 0 to 1, while higher scores indicate higher stemness (less differentiation) and vice versa.

The R package Seurat v4.0.6 was used to identify differentially expressed genes of malignant cells in each dataset. Genes with the log-fold change (logFC) ≥ 0.25 and false discovery rate (FDR) < 1e−05 were considered as differentially up-regulated genes of malignant cells in each dataset [62].

### Anti-tumor immunity and pathway analysis

Over Representation Analysis (ORA) was conducted to determine whether known biological functions or processes are enriched in Stem.Sig [67]. The R package *clusterProfiler* v4.2.1 was used to perform ORA and visualize the results [68].

Gene set variation analysis (GSVA) was used to calculate the Stem.Sig scores and relative enrichment of other gene signatures and biological pathways across sample populations. The R package *GSVA* v1.42.0 was applied to perform GSVA [69].

We further evaluated the correlation between Stem.Sig and tumor-infiltrating leukocytes (TILs)/immune-related genes (IRGs) in TCGA cohort. Immune-related genes and their functional classifications were obtained from Thorsson et al. [66]. The R package *MCP-counter* v1.1.0 was utilized to estimate the abundance of tumor-infiltrating leukocytes [70].

### Clinical outcomes

The primary clinical outcomes were objective response rate (ORR) and overall survival (OS). ORR was assessed using Response Evaluation Criteria in Solid Tumors (RECIST) version 1.1 in all cohorts [71], except cohort Hugo 2016 [59], whose ORR was assessed using immune-related RECIST (irRECIST). Patients were divided into two groups according to their response status: complete response (CR) and partial response (PR) as responders, or stable disease (SD) and progressive disease (PD) as non-responders.

### Derivation of predictive model for ICI response

#### Dataset

Top five ICI RNA-Seq cohorts with most patients were combined to form a large cohort (*n*= 772), including RCC (*n*=181), UC (*n*=348), and SKCM (*n*=243). These five cohorts are Braun 2020 RCC [52], Mariathasan 2018 UC [53], Liu 2019 SKCM [54], Gide 2019 SKCM [55], and Riaz 2017 SKCM [56]. We used ComBat method to remove the batch effect of different ICI RNA-Seq cohorts [72]. Then we randomly split this combined cohort into two datasets: training set (80%, *n* = 618) and validation set (20%, *n*=154). The other five ICI RNA-Seq cohorts [57–61] were consolidated as an independent testing set (*n* = 149).

#### Model training and parameter tuning

We trained the ICI response classification model with Stem.Sig, using seven common machine learning (ML) algorithms, including support vector machine (SVM), Naïve Bayes (“NB”), random forest (“RF”), k-nearest neighbors (“KNN”), AdaBoost Classification Trees (“AdaBoost”), boosted logistic regressions (“LogiBoost”), and cancerclass [73, 74]. For each ML algorithm with parameters except cancerclass, fivefold cross-validation (CV) was adopted for hyperparameter tuning to optimize the performance of the model. To ensure robustness, we repeated the optimization process 10 times with different random seeds for each single resampling [75]. As for cancerclass which does not require parameters, we trained the model using the entire training set directly.

#### Model validation and independent testing

We had seven models derived from the training set, using different ML algorithms. Then we applied these models to the validation set and compared their results. The model with the best performance was chosen as the final Stem.Sig model. To evaluate the predictive value of the final model, we applied it to the testing set.

#### Comparing Stem.Sig with other predictive gene signatures

To further evaluate the predictive value of Stem.Sig, we compared Stem.Sig with other ICI response signatures reported previously, including six pan-cancer signatures (INFG.Sig [76], T.cell.inflamed.Sig [76], PDL1.Sig [77], LRRC15.CAF.Sig [78], NLRP3.Sig [79], and Cytotoxic.Sig [80]) and seven melanoma-specific signatures (CRMA.Sig [81], IMPRES.Sig [7], IPRES.Sig [82], TcellExc.Sig [14], ImmmunCells.Sig [83], IMS.Sig [84], and TRS.Sig [85]). Pan-cancer signatures were compared with Stem.Sig in the testing set regarding the performance of ICI response prediction. As for melanoma-specific signatures, we compared their performance with Stem.Sig using melanoma patients from the testing set (Hugo 2016 [59] and Van Allen 2015 [60]). Codes and algorithms for the 13 aforementioned signatures were derived from their original studies, such as ssGSEA for NLRP3.Sig [79], cancerclass for ImmuneCell.Sig [83], overall expression for TcellExc.Sig [14], and so on. Details of these signatures and their corresponding algorithms can be found in Additional file 1: Table S5.

### Statistical analysis

Statistical analyses were performed using R v4.1.1 (https://www.r-project.org). Comparison of CytoTRACE scores between response and non-response subgroups was analyzed by two-sided Wilcoxon tests. We used Spearman correlation to evaluate the association between Stem.Sig and biological pathways or immune features. Benjamini-Hochberg procedure (B-H) was applied to calculate FDR. The R package *caret* v6.0-90 and *cancerclass* v1.34.0 were used for model training, validation, and testing [74]. The receiver operating characteristic (ROC) curve was used and a larger area under the ROC curve (AUC) indicated a better predictive performance An AUC of 0.9–1.0 is considered excellent, 0.8–0.9 very good, 0.7–0.8 good, 0.6–0.7 sufficient, 0.5–0.6 bad, and less than 0.5 considered not useful [86]. Patients predicted by the final model as “NR” and “R” were categorized into high-risk and low-risk subgroups for survival analysis. The association between the Stem.Sig-based risk grouping and OS was analyzed by Cox proportional hazards regression analysis. Further survival analysis of individual cohort from testing set was adjusted for available confounding factors, including TMB, tumor purity, sex, and age.

## Results

### Cancer stemness is associated with ICI resistance

**Fig. 1:**
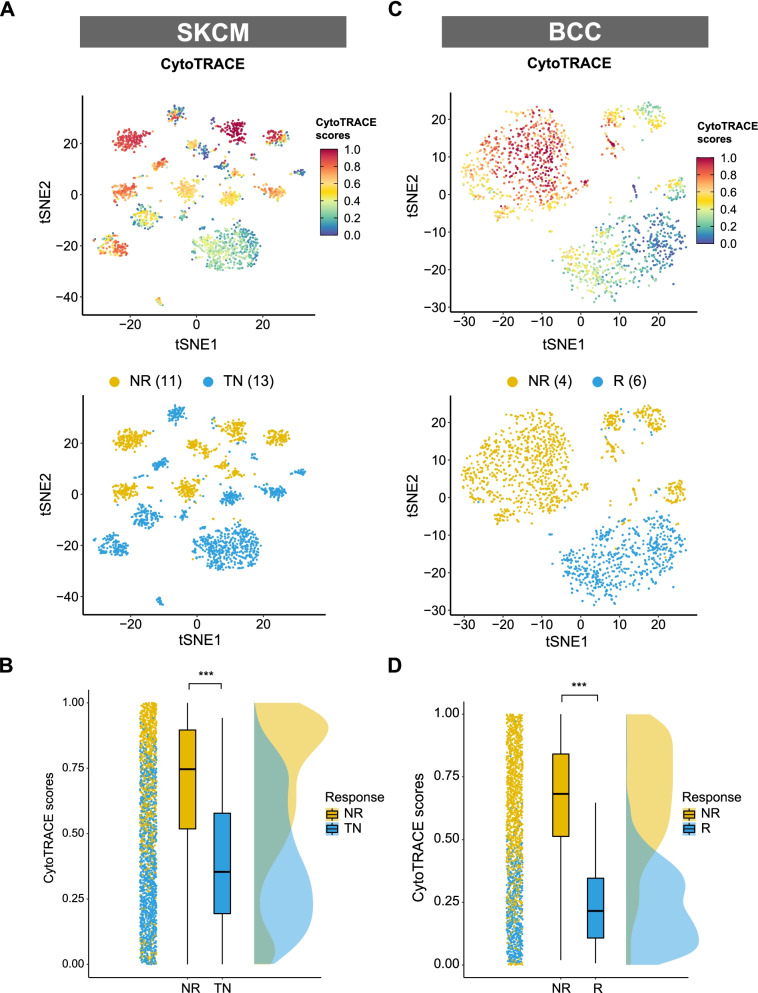
Identification and validation of a negative association between cancer cell stemness and ICI outcomes. **A**, **C** t-Distributed Stochastic Neighbor Embedding (tSNE) plot of malignant cells from SKCM or BCC. Top tSNE plots depicting the distribution of CytoTRACE scores among malignant cells. Dark-green indicates lower scores (low stemness) while dark-red indicates higher scores (high stemness). Bottom tSNE plots label the malignant cells by response phenotype. **B**, **D** raincloud plot of CytoTRACE scores by response phenotype (NR vs. TN) in SKCM cohort or by response phenotype (NR vs. R) in BCC cohort. The center of the box plot are median values, the bounds of the box are 25% and 75% quantiles (Wilcoxon test; *** *P* < 0.001). Abbreviation: NR, non-responders; R, responders; TN, treatment naïve patients.

A previous published ICI SKCM cohort with scRNA-seq data was firstly employed to evaluate the association between cancer stemness and ICI outcomes [14]. After removing patients without malignant cells data, we adopted 24 patients from this cohort, consisting of 11 non-responders (NR) and 13 treatment-naïve patients (TN). Ideally, it is better to compare the cancer stemness between responders (R) and non-responders. However, data of responders was not available in this cohort. Given that treatment naïve patients likely include both potential responders and non-responders, comparison of stemness was conducted between NR and TN as previously described [14]. As shown in Fig. 1A, cancer cells with high stemness were enriched in the NR subgroup. Further analysis showed that tumors from the NR subgroup had a significantly higher level of stemness (*P* < 0.001, Fig. 1B), indicating that cancer stemness is negatively associated with ICI outcomes. Another ICI cohort with a different cancer type (BCC) was employed to validate this finding [15]. In the BCC cohort, tumor stemness of 4 non-responders was compared to that of 6 responders. We found a more prominent gap of stemness level between NR and R subgroups in the BCC cohort (*P* < 0.001, Fig. 1C and D).

### Development of Stem.Sig through pan-cancer scRNA analysis

**Fig. 2:**
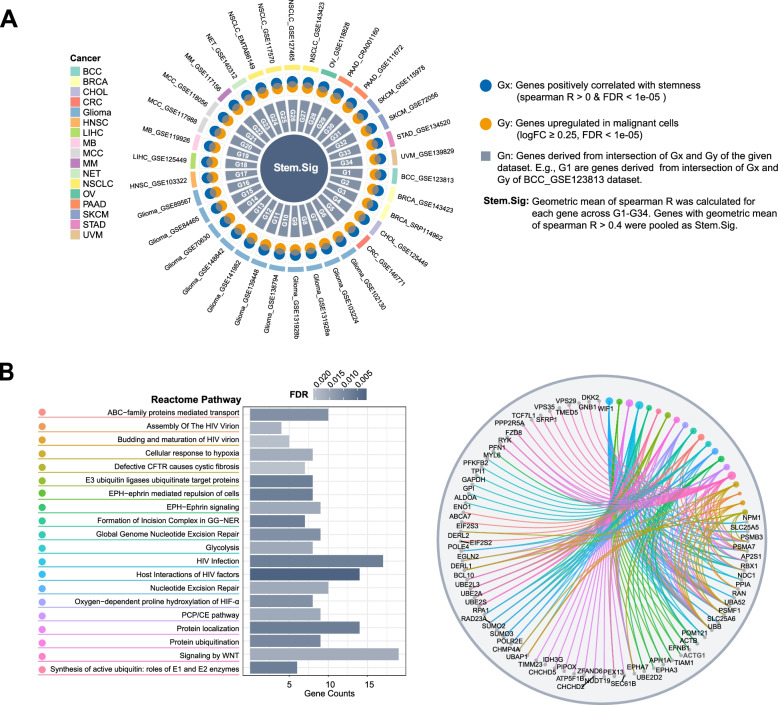
Development and description of stemness signature. **A** Circos plot depicting the development of Stem.Sig. **B** Pathway enrichment analysis of genes in Stem.Sig. The bar plot showed the top 20 enriched Reactome pathways. The cnetplot presented the network of specific genes from these pathways. Colored points referred to the corresponding pathways. Abbreviation: CFTR, cystic fibrosis transmembrane conductance regulator; GG-NER, global genomic nucleotide excision repair; HIF, hypoxia-inducible factor; PCP, planar cell polarity; CE, convergent extension

As cancer stemness is significantly associated with ICI resistance, we hypothesized that a Stem.Sig reflecting the stemness level of the tumor may help in the prediction of ICI efficacy. Therefore, 34 scRNA-Seq datasets were employed to develop the Stem.Sig (Fig. 2A; Additional file 1: Table S6). We performed Spearman correlation analysis between gene expression level and CytoTRACE scores for malignant cells among pan-cancer scRNA datasets. Genes that were positively correlated with CytoTRACE scores (Spearman *R* > 0 and FDR < 1e−05) were regarded as Gx. Genes that were differentially up-regulated in malignant cells were regarded as Gy. To obtain up-regulated tumor-specific genes that were positively associated with stemness, Gx and Gy were intersected to give rise to Gn for each dataset [14]. For example, G_1_ consisted of genes derived from the intersection of Gx and Gy in the first scRNA-Seq dataset. Geometric mean of Spearman *R* was calculated for each gene across G1–G34. Finally, genes with geometric mean of Spearman *R* > 0.4 (moderate to strong correlation) were pooled as Stem.Sig [87].

We investigated the biological functions that were over-represented in Stem.Sig (Fig. 2B). The enriched pathways mainly comprise processes involving hypoxia, glycolysis, ubiquitination, EPH-ephrin signaling, WNT signaling, and nucleotide excision repair (NER). Specific genes of these pathways were shown in the cnetplot of Fig. 2B. Some genes have been reported to be associated with unfavorable outcomes of immunotherapy, such as EPHA3, EPHA7, ENO1, ACTG1, DKK2, NPM1, and BCL10 [6, 88–92].

### Analysis of the potential links between Stem.Sig and immune suppression using pan-cancer TCGA cohort

**Fig. 3:**
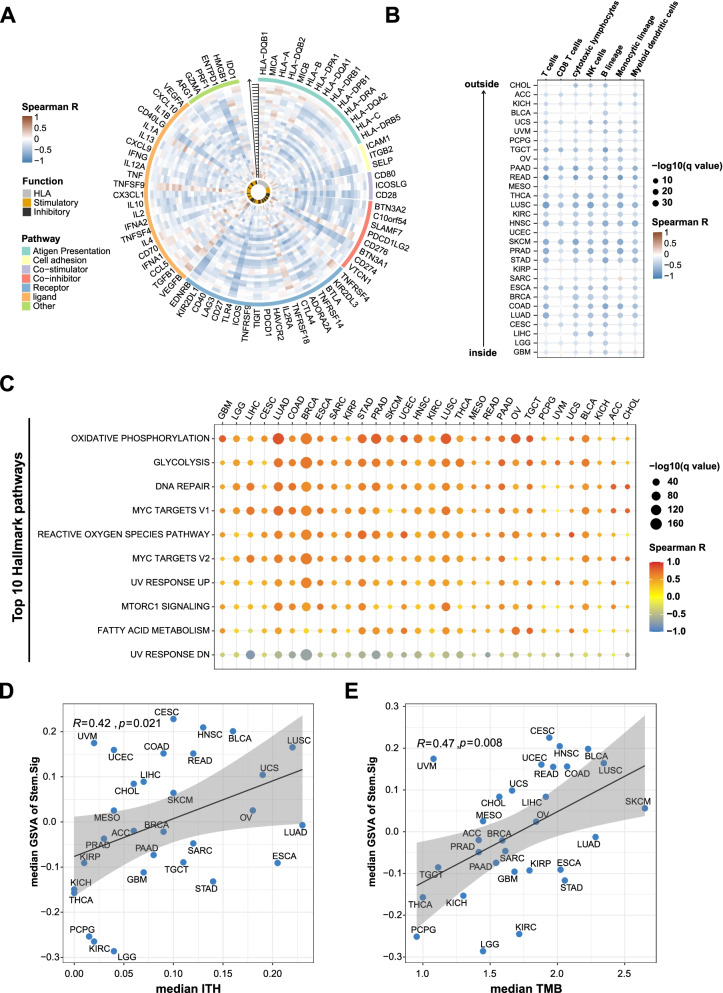
Analysis of the potential links between Stem.Sig and immune resistance using pan-cancer TCGA cohort. **A** Circos plot depicting the correlation between Stem.Sig and the expression level of immune-related genes across multiple cancer types. From inside to outside of the circos plot, the vertical axis with a black arrow indicated different cancer types, which were annotated by the y axis of plot B. **B** Heatmap depicting the correlation between Stem.Sig and the infiltration of immune cells across multiple cancer types. **C** Heatmap depicting the correlation between Stem.Sig and the Top 10 Hallmark pathways. **D** Correlation of median Stem.Sig and median TMB of each cancer type. **E** Correlation of median Stem.Sig and median ITH of each cancer type. GSVA scores were calculated to estimate the expression level of Stem.Sig for each sample

First, we performed a thorough analysis of Stem.Sig and 75 immune-related genes [66]. A general negative association was observed between Stem.Sig and expression level of immune-related genes across 30 different cancer types (Fig. 3A). Then we evaluated the infiltration status of immune cells to better characterize the tumor immune microenvironment (TIME). Tumors with high Stem.Sig had decreased cytotoxic immune cells, including CD8^+^ T cells, NK cells and macrophages (Fig. 3B). Taken together, these results indicated that Stem.Sig was negatively associated with anti-tumor immunity.

Secondly, we analyzed the enrichment of hallmark pathways regarding the expression level of Stem.Sig to investigate whether immunosuppressive biological functions were upregulated in high Stem.Sig tumors. Metabolic pathways, DNA repair, and MYC signaling were found to be enriched in tumors with high Stem.Sig(Fig. 3C). All of these pathways contributed to the poor immune response according to previous studies [93–95].

Furthermore, we investigated the association between Stem.Sig and ITH, a stemness-associated feature that mediates immunosuppression [12]. As expected, ITH were positively correlated with Stem.Sig across cancer types. (*R* = 0.42, *P* = 0.021, Fig. 3D). Same analysis was applied to TMB, a well-known immune-relevant factor. Interestingly, a similar positive association was detected between Stem.Sig and TMB as well (*R* = 0.47, *P* = 0.008, Fig. 3E). High TMB indicates better immune response, while high Stem.Sig dose the opposite. To better elucidate the correlation of anti-tumor immunity with both Stem.Sig and TMB, we further divide patients into four subgroups: high Stem.Sig/high TMB (HSHT), high Stem.Sig/low TMB (HSLT), low Stem.Sig / high TMB (LSHT), and low Stem.Sig / low TMB (LSLT). Median GSVA score of Stem.Sig and median TMB were used as thresholds for grouping. Then we compared the abundance of immune cells among these four subgroups. Interestingly, LSHT was found with the highest level of cytotoxic lymphocytes (*P* < 0.001), while HSLT with the lowest level (*P*<0.001). High cancer stemness (HS) could promote immune resistance and evasion, while low TMB level (LT) is associated with decreased anti-tumor immunity due to lack of antigenicity. As expected, decreased infiltration of cytotoxic lymphocytes was detected for both HS and LT groups (*p* < 0.001, Additional file 2: Fig.S1 A and B). It is reasonable that the coexistence of these two factors (HSLT) may result in a TIME with the least infiltration of cytotoxic lymphocytes. On the contrary, LSHT could lead to the most abundant CTLs in the TIME. However, the anti-tumor immunity of the other two groups (HSHT and LSLT) seems to be more controversial than the aforementioned groups (HSLT, LSHT), since HSHT and LSLT both have an immune-suppressed (HS or LT) factor and an immune-promoted (LS or HT) factor. Further subgroup analysis found a higher level of cytotoxic lymphocytes in LSLT than in HSHT (*p* < 0.001, Additional file 2: Fig.S1 C). In conclusion, the order of anti-immunity from highest to lowest is: LSHT > LSLT > HSHT > HSLT (all *p* < 0.001, Additional file 2: Fig.S1 C). Therefore, tumors with low Stem.Sig presented with significantly better anti-tumor immunity than those with high Stem.Sig regardless of TMB level.

### Immunotherapy outcome prediction by Stem.Sig

**Fig. 4:**
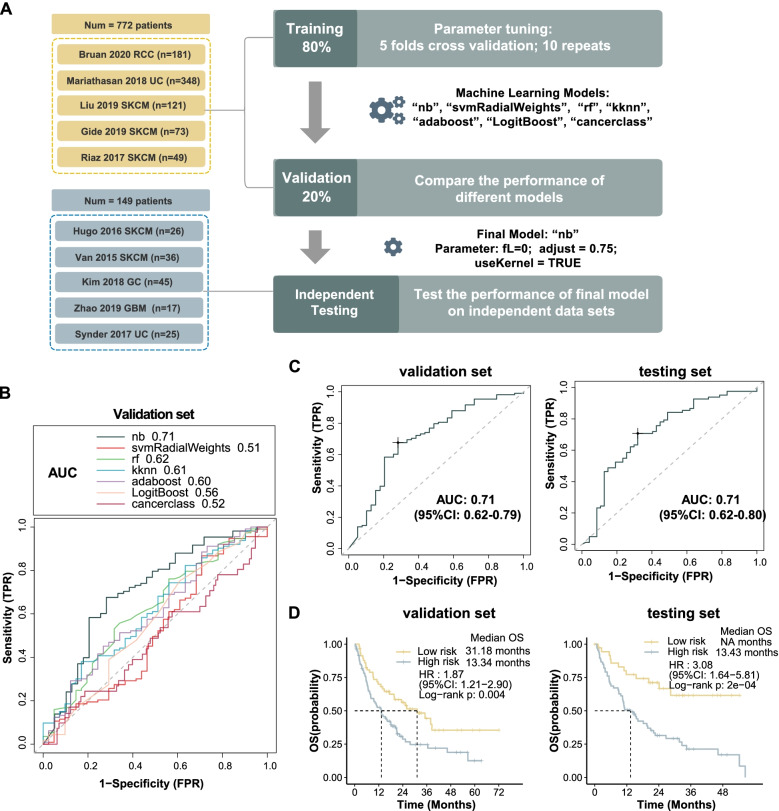
Prediction of ICI outcomes using Stem.Sig. **A** Flow chart of training, validating, and testing the Stem.Sig model constructed using machine learning process. In the training set, we applied 10-time repeated 5-fold cross-validation for parameters tuning of different machine learning algorithms. In the validation set, Naïve Bayes algorithm with best AUC was kept as the final Stem.Sig model. (parameter: fL=0; adjust = 0.75; useKernel = TRUE). **B** Comparison of multiple ROC plot depicting the performance of different machine learning algorithms in the validation set. **C** ROC plot depicting the performance of the final Stem.Sig model in validation and testing cohort. **D** Kaplan-Meier curves comparing OS between High-risk and Low-risk patients in validation and testing set. “NR” and “R” predicted by the final Stem.Sig Model was defined as “High-risk” and “Low-risk” patients respectively. HR were calculated by Cox proportional hazards regression analysis. Abbreviation: TPR, true positive rate; FPR, false positive rate; AUC, area under the curve; HR, hazard ratio; CI, confidence intervals

To investigate the predictive value of Stem.Sig, we collected bulk RNA-Seq data and clinical information from 10 ICI cohorts. Pre-treatment samples of these cohorts were curated and analyzed. Patients received anti-PD(L)-1, anti-CTLA-4, or anti-PD(L)-1 plus anti-CTLA-4. All these 10 cohorts were split into 3 data set: training set (*n*=620), validation set (*n*=154), and testing set (*n*=149). The flow chart of the analysis process was shown in Fig. 4A. Firstly, we trained the model with seven different machine learning algorithms and applied 10-time repeated 5-fold cross-validation for parameter optimization of each model. After training, we harvested seven models. Then, we evaluated and compared the AUC of these models in the validation cohort. Naïve Bayes model achieved the highest AUC of 0.71 and was selected as Stem.Sig model (Fig. 4B). For further assessment of the Stem.Sig model, we applied it to the independent testing set to predict ICI response and observed a same AUC of 0.71 (Fig.4C).

To evaluate whether the Stem.Sig model can predict overall survival, we divided ICI-treated patients into low-risk and high-risk subgroups based on the predicted “R” and “NR” respectively. The Kaplan-Meier analysis of OS was shown in Fig. 4D. Low-risk group achieved a significantly longer overall survival in training, validation, and testing sets (all log-rank *p* < 0.01). In the validation cohort, high-risk patients predicted by the Stem.Sig model had a median OS of only 13.3 months, compared to 31.2 months of low-risk patients (HR: 1.87; 95%CI: 1.21–2.90). In the testing set, a similar median OS of 13.4 months was observed in high-risk patients, while low-risk ones had not reached the median OS (HR: 3.08; 95%CI: 1.64–5.81).

We performed subgroup analysis for five individual cohorts that contribute to the testing set. Regarding ICI response prediction, AUC ranged from 0.62 to 0.81 among these cohorts (Additional file 2: Fig.S2A). Van Allen 2015 SKCM achieved a favorable AUC of 0.81 (95%CI: 0.66−0.95), followed by Synder 2017 UC (AUC: 0.80; 95%CI: 0.61−0.99). Compared to other cohorts, Zhao 2019 GBM presented with the lowest AUC of 0.62 (95%CI: 0.33−0.91). In survival analysis, Kim 2018 GC was removed due to a lack of OS data. For the other four cohorts, we observed a HR ranged from 1.73 to 4.05 in high-risk patients predicted by the Stem.Sig model (Additional file 2: Fig.S2B). After adjusting available confounding factors, significant survival benefits were still found in Van Allen 2015 SKCM (adjusted *p* = 0.02) and Synder 2017 UC (adjusted *p* = 0.02), while the other two cohorts showed only numerical survival differences. It is possibly due to the limited sample size.

**Fig. 5:**
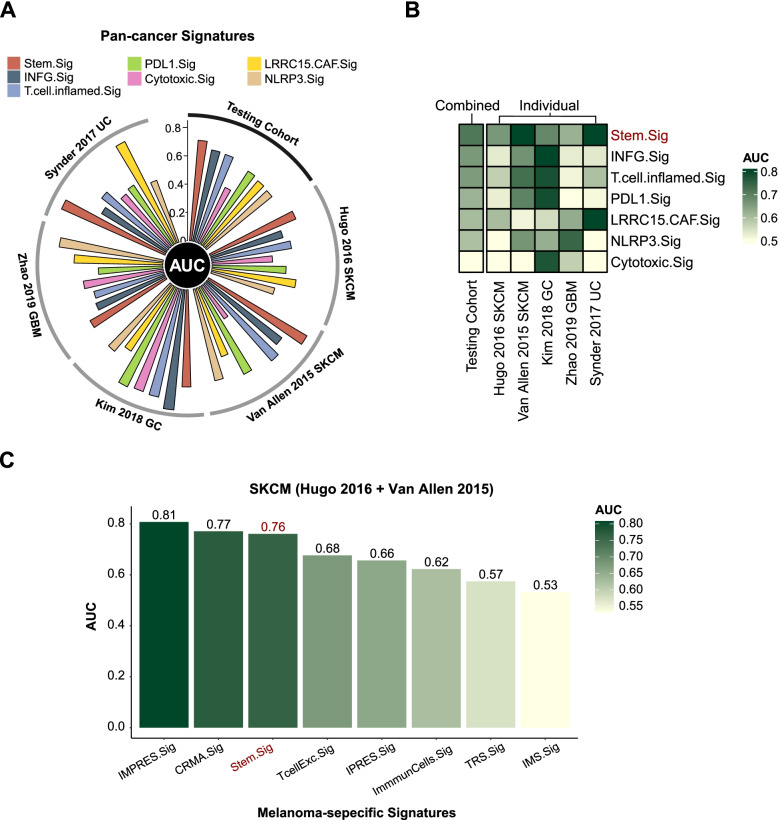
Comparing AUC of Stem.Sig with other predictive gene signatures. **A** Circos plot depicting the performance of pan-cancer signatures in the testing set. The vertical axis indicated AUC values. Testing set comprises five different cohorts, including Hugo 2020 SKCM, Van Allen 2015 SKCM, Kim 2018 GC, Zhao 2019 GBM, Synder 2017 UC. **B** Heatmap comparing the predictive value of Stem.Sig and other pan-cancer signatures. Different signature rows were ordered by their AUC in the testing set. From top to bottom, Stem.Sig ranked first while Cytotoxic.Sig ranked last. **C** Bar plot depicting the AUC values of Stem.Sig and other melanoma-specific signatures in the SKCM cohort (Hugo 2016 + Van Allen 2015).

We further compared the performance of Stem.Sig with previous well-established predictive gene signatures. Compared with pan-cancer signatures (INFG.Sig [76], T.cell.inflamed.Sig [76], PDL1.Sig [77], LRRC15.CAF.Sig [78], NLRP3.Sig [79], and Cytotoxic.Sig [80]), Stem.Sig showed best performance in the testing set with an AUC of 0.71, followed by INFG.Sig with an AUC of 0.66 (Fig. 5A). Most of these pan-cancer signatures showed ideal performance in only one or two cohorts. For example, AUC of INFG.Sig reached 0.85 in Kim 2018 GC and 0.67 in Van Allen 2015 SKCM, but it decreased to 0.53–0.54 in the other three cohorts (Additional file 1: Table S7). However, Stem.Sig achieved sufficient to very good performance in all cohorts, covering four cancer types: SKCM, GBM, UC, and GC, which further stresses its potential as a predictive model of ICI response in a pan-cancer manner (Fig. 5B). Compared with melanoma-specific signatures (CRMA.Sig [81], IMPRES.Sig [7], IPRES.Sig [82], TcellExc.Sig [14], ImmmunCells.Sig [83], IMS.Sig [84], and TRS.Sig [85]), Stem.Sig remained in the top 3 with an AUC of 0.76 in prediction of ICI response regarding melanoma patients. IMPRES.Sig and CRMA.Sig showed a slightly better AUC of 0.81 and 0.77 than Stem.Sig.

### Exploration of potential therapeutic targets from Stem.Sig using CRISPR screen data

**Fig. 6:**
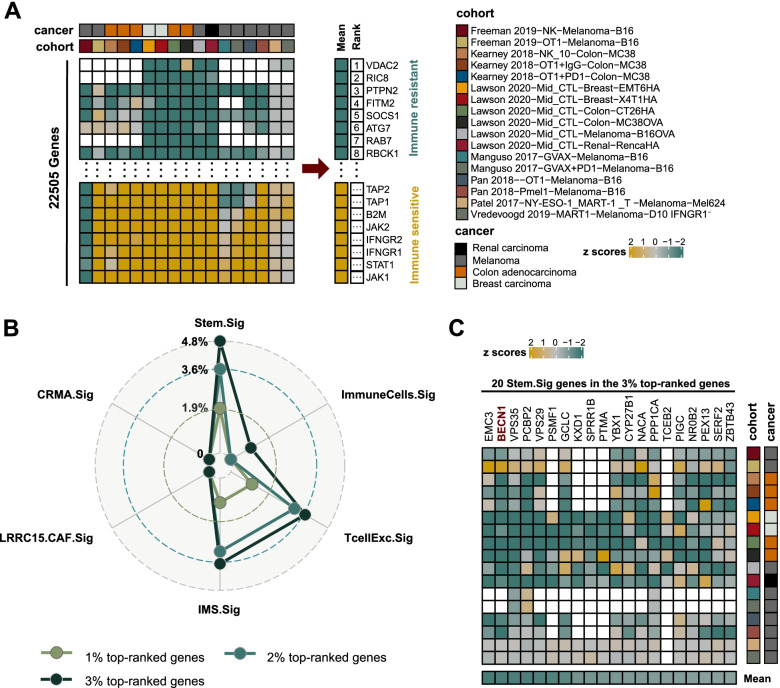
Exploration of potential treatment targets from Stem.Sig using CRISPR screening data. **A** Ranking of genes based on their knockout effects on anti-tumor immunity across 17 CRISPR datasets. Negative (positive) *z* scores indicated better (worse) immune response after knockout of a specific gene. Genes were ranked according to their mean *z* scores. Top-ranking genes were associated with immune resistance. Blank squares in the heatmap referred to missing values of gene data from the corresponding cohort. **B** Radar plot comparing the percentage of top-ranked genes for Stem.Sig and other predictive signatures. **C** Heatmap depicting *z* scores of 20 Stem.Sig genes in the 3% top-ranked genes across different CRISPR datasets

We systemically collected immune response data of knockout genes from seven CRISPR cohorts, which were further divided into 17 datasets according to the model cells and treatment conditions used in these CRISPR cohorts. Totally, there were 22,505 genes recorded by these CRISPR datasets. We ranked genes based on their mean *z* scores. Top-ranked genes were immune-resistant genes, which may promote anti-tumor immunity after knockout. Bottom-ranked genes were immune-sensitive genes, which may suppress anti-tumor immunity after knockout. The process of gene ranking was shown in Fig. 6A. Among all 22,505 genes, the number of 1%, 2%, and 3% top-ranked genes was 225, 450, and 675, respectively. Next, we calculated the percentage of top-ranked genes that were presented in Stem.Sig and previous immune-resistant signatures, including TcellExc.Sig, ImmuneCells.Sig, IMS.Sig, LRRC15.CAF.Sig, and CRMA.Sig (except IPRES.Sig, which comprises 73 genetic pathways instead of individual genes) [14, 78, 81, 83, 84]. Stem.Sig, TcellExc.Sig, IMS.Sig, and ImmuneCells.Sig were the only four gene sets that had genes ranked in the top 3%. As expected, Stem.Sig had the highest percentage of top-ranked genes than other signatures (Fig. 6B). Immune-resistant genes (3% top-ranked genes) were significantly over-represented in Stem.Sig (*P*=0.03; Fisher’s exact test). There were 20 genes of Stem.Sig that were ranked in the top 3%, including EMC3, BECN1, VPS35, PCBP2, VPS29, PSMF1, GCLC, KXD1, SPRR1B, PTMA, YBX1, CYP27B1, NACA, PPP1CA, TCEB2, PIGC, NR0B2, PEX13, SERF2, and ZBTB43. Immune-resistant features of these stemness-associated genes were validated by multiple independent CRISPR datasets (Fig. 6C), which may serve as potential therapeutic targets in synergy with ICB.

## Discussion

Although the mechanism between cancer stemness and anti-tumor immunity has been widely explored [10, 12, 96, 97], direct clinical evidence on the association of stemness and ICI response has not been reported. Here we utilized CytoTRACE to evaluate the stemness level of individual malignant cells and uncovered the inverse correlation between stemness and ICI outcomes, supported by the results from two ICI scRNA-Seq cohorts of SKCM and BCC [14, 15]. CSCs have been found in virtually all solid tumors [10]. Motivated by these observations, we hypothesized that the negative association between stemness and ICI efficacy generally existed across various cancers. Therefore, a large-scale comprehensive analysis was performed to identify over-expressed genes in malignant cells that significantly correlated with increased stemness. These genes formed a pan-cancer stemness signature, namely, Stem.Sig. We carefully validated the predictive value of Stem.Sig. Remarkably, Stem.Sig achieved better performance of predicting ICI response than previous predictive signatures across multiple independent ICI cohorts with bulk RNA-Seq data [52–61]. This study is the first report to demonstrate the robust link between stemness and ICI outcomes through a comprehensive analysis of large-scale data. Most importantly, we constructed a gene expression signature, Stem.Sig, that successfully predicts response to immunotherapy across multiple cancer types.

We found that Stem.Sig genes were enriched in the following biological functions: hypoxia, glycolysis, ubiquitination, nucleotide excision repair, EPH-ephrin signaling, and WNT signaling. WNT signaling is the key pathway that drives self-renewal of CSCs and maintains cancer stemness [98]. Hypoxia causes an increase in transcription factors (e.g., OCT4, SOX2, c-myc, and Nanog) which contribute to the sustenance of CSCs [99]. Anaerobic glycolysis is the distinct metabolic hallmark of stem cells [100]. Ubiquitination-mediated transcriptional regulatory network is essential in the maintenance of the stemness and pluripotency of stem cells [101]. Nucleotide excision repair (NER) is a major DNA repair pathway, which preserves genome integrity of cancer stem cells as to overcome stressful conditions [93]. Activity of EPH-ephrin signaling, as the largest family of receptor tyrosine kinases, is found enhanced in CSCs [102]. In our previous study, nonsynonymous somatic mutations of EPHA3 and EPHA7 was found associated with improved ICI efficacy [6]. It is reasonable that elevated EPH-ephrin signaling may contribute to the immunosuppressive features of CSCs. Furthermore, we evaluated the correlation between Stem.Sig and twelve previously identified stemness signatures [12]. As expected, Stem.Sig was found positively associated with these stemness signatures across different cancer types (Additional file 2: Fig. S3). Our results were in line with previous studies and suggested that Stem.Sig encompasses genes that robustly and specifically correlate with cancer stemness.

TCGA pan-caner transcriptomic analysis revealed a consistently down-regulated expression of immune-related genes and reduced infiltration of immune cells in tumors with high Stem.Sig level across different cancer types. Interestingly, a negative association between B cells and Stem.Sig was also observed. B cells could favorably affect ICI response via tertiary lymphoid structure (TLS), and hence we analyzed the relationship between TLS and Stem.Sig [103]. TLS scores were found inversely associated with Stem.Sig (Additional file 2: Fig. S4). Further analysis also revealed an up-regulation of some immune-relevant biological functions, including metabolism, DNA repair, and MYC signaling. Acquisition of hypermetabolic phenotype is an evolving mechanism that mediates immune evasion [94]. Enhanced DNA-repair capacity prepared malignant cells for unfriendly environments [93]. Increased MYC signaling suppresses immune response by elevating expression of PD-L1 and CD47 [95]. Tumors with high Stem.Sig presented with substantially immunosuppressive features, which corroborate the predictive value of Stem.Sig.

Also, we observed a positive correlation between Stem.Sig and both TMB and ITH, which is similar to the results of Miranda et al. [12]. It is noteworthy that high TMB is associated with high stemness. Although TMB is a well-recognized ICI biomarker, there is still a significant number of patients with high TMB fail to response to ICI [104]. Our stratified analysis revealed a significantly negative correlation between Stem.Sig and anti-tumor immunity in both low TMB and high TMB tumors. Cancer stemness can be a reasonable explanation of the immune resistance of high TMB tumors, which further stressed the importance of Stem.Sig as a predictive ICI biomarker.

Stem.Sig is a novel biomarker that is capable of predicting ICI response effectively and distinguishing patients with survival benefits successfully. We further compared Stem.Sig with other state-of-the-art signatures, including six pan-cancer signatures [76–80] and seven melanoma-specific signatures [7, 14, 81–85]. Stem.Sig outperformed pan-cancer signatures with better generalization and achieved an overall favorable performance in different cohorts across multiple cancer types. Compared with melanoma-specific signatures, Stem.Sig ranked top 3 and achieved a competitive AUC of 0.76.

Biomarker research is not only for improving patient selection but also for combination strategies that can overcome immune resistance. Considering such a robust link between Stem.Sig and ICI outcomes, we used CRISPR datasets to explore potential drug targets from Stem.Sig. We ranked genes based on their relevance to immune response and harvested the most immune-resistant Stem.Sig genes. For example, BECN1 is among the top-ranked Stem.Sig genes to render the TIME resistant to ICI. BECN1 plays a central role in autophagy, which is essential for self-renewal of CSCs and the maintenance of cancer stemness [105]. Targeting BECN1 can induce expression of CCL5, promote infiltration of NK cells, and thus improve antitumor immune response [106]. Top-ranked Stem.Sig genes, such as EMC3, BECN1, VPS35, and PCBP2, showed improved immune response after knockout in melanoma, renal carcinoma, breast carcinoma, and colon adenocarcinoma from multiple CRISPR datasets. These stemness-associated genes could be potential therapeutic targets for various cancer types. Further research of these top-ranked Stem.Sig genes would help to develop a combined strategy of immunotherapy.

Our study has some limitations. First, there were only treatment naïve patients and non-responders from GSE115978 [14]. Comparison of the cancer stemness was conducted between non-responders and treatment naïve patients. Considering the average response rate of melanoma is 30–40%, a considerable proportion of treatment naïve patients would probably not response to ICI. Theoretically, the difference between TN and NR is smaller than that of R and NR, since TN is a mixture of NR and R. However, a significant difference of stemness level still existed between NR and TN in this study, which indicates an even greater gap between NR and R. And this was confirmed by analysis of another scRNA-Seq ICI cohort, GSE123813 [15]. Secondly, some clinical annotation data (e.g., sex/age/tumor stage/TMB/ITH) was unavailable in some RNA-Seq ICI studies for multivariate cox regression analysis of overall survival. Thirdly, the 10 RNA-Seq ICI cohorts adopted in our studies only cover five cancer types (GC, SKCM, RCC, UC, and GBM). The consistent negative association between Stem.Sig and anti-tumor immunity across 30 cancer types can compensate this to some degree. Still, the predictive value of Stem.Sig in a pan-caner setting needs to be verified by future prospective ICI trials.

## Conclusions

We provided the first solid clinical evidence that cancer stemness was associated with immunotherapy resistance. Using pan-cancer analysis of single-cell transcriptomic data, we developed a gene expression signature, Stem.Sig, which outweighs other well-established signatures in predicting ICI outcomes across multiple cohorts. Further exploration of Stem.Sig also revealed some potential therapeutic targets. Our study demonstrates a promising solution for patient selection in immunotherapy and sheds light on tackling ICI resistance through targeting cancer stemness to boost anti-tumor immunity.

## Supplementary Information

**Additional file 1: Table S1.** Characteristics of ICIs scRNA cohorts. **Table S2.** List of scRNA datasets applied to develop Stem.Sig. **Table S3.** List of immunotherapy cohorts used in this study. **Table S4.** List of CRISPR datasets. **Table S5.** List of predictive gene expression signatures for immunotherapy. **Table S6.** Gene list of Stem.Sig. **Table S7.** Comparison of AUC of previous signatures in testing cohort.

**Additional file 2: Figure S1.** Boxplots depicting the correlation of immune cells infiltration with Stem.Sig and TMB. **Figure S2.** Subgroup analysis of testing set. **Figure S3.** Correlation plot of Stem.Sig and other stemness signatures. **Figure S4.** Association Stem.Sig and expression level of TLS-related genes.

## Data Availability

All data used in this study are publicly available as described in the Method section. The web links or unique identifiers for public cohorts/datasets are described in the paper. Source codes and all supplementary data used to generate the results were deposited on figshare (10.6084/m9.figshare.17654633) [107].

## Abbreviations

AUC: Area under the ROC curve
BCC: Basal cell carcinoma
B-H: Benjamini-Hochberg procedure
BRCA: Breast cancer
CHOL: Cholangiocarcinoma
CR: Complete response
CRC: Colorectal cancer
CSCs: Cancer stem cells
DLBC: Large B cell lymphoma
FDR: False discovery rate
GSVA: Gene set variation analysis
HNSC: Head and neck cancer
HSHT: High Stem.Sig and high TMB
HSLT: High Stem.Sig and low TMB
ICI: Immune checkpoint inhibitor
IRGs: Immune-related genes
ITH: Intra-tumoral heterogenicity
LAML: Acute myeloid leukemia
LIHC: Liver hepatocellular carcinoma
logFC: Log-fold change
LSHT: Low Stem.Sig and high TMB
LSLT: Low Stem.Sig and low TMB
MB: Medulloblastoma
MCC: Merkel cell carcinoma
MM: Multiple myeloma
NER: Nucleotide excision repair
NET: Neuroendocrine tumor
NSCLC: Non-small cell lung cancer
NR: Non-responders
ORA: Over Representation Analysis
ORR: Objective response rate
OS: Overall survival
OV: Ovarian serous cystadenocarcinoma
PAAD: Pancreatic adenocarcinoma
PD: Progressive disease
PR: Partial response
R: Responders
RCC: Renal cell carcinoma
RECIST: Response Evaluation Criteria in Solid Tumors
ROC: The receiver operating characteristic
scRNA-Seq: Single-cell RNA sequencing
SD: Stable disease
SKCM: Skin cutaneous melanoma
STAD: Stomach adenocarcinoma
TCGA: The Cancer Genome Atlas
THYM: Thymoma
TILs: Tumor-infiltrating leukocytes
TMB: Total mutational burden
TN: Treatment-naïve patients
UVM: Uveal melanoma
WES: Whole exome sequencing
