# Supplementary material for: Integrated analysis of single-cell and bulk RNA sequencing data reveals a pan-cancer stemness signature predicting immunotherapy response
Source: Genome Med. 2022 Apr 29;14:45. doi: 10.1186/s13073-022-01050-w (PMC9052621; doi:10.1186/s13073-022-01050-w)
Supplement: Supplementary file 1 — Additional file 1: Table S1. Characteristics of ICIs scRNA cohorts. Table S2. List of scRNA datasets applied to develop Stem.Sig. Table S3. List of immunotherapy cohorts used in this study. Table S4. List of CRISPR datasets. Table S5. List of predictive gene expression signatures for immunotherapy. Table S6. Gene list of Stem.Sig. Table S7. Comparison of AUC of previous signatures in testing cohort. [file 13073_2022_1050_MOESM1_ESM.pdf]

Supplementary Material

Table S1. Characteristics of ICIs scRNA cohorts. 2

Table S2. List of scRNA datasets applied to develop Stem.Sig. 3

Table S3. List of immunotherapy cohorts used in this study. 4

Table S4. List of CRISPR datasets 5

Table S5. List of predictive gene expression signatures for immunotherapy 6

Table S6. Gene list of Stem.Sig. 7

Table S7. Comparison of AUC of previous signatures in testing cohort. 8

Table S1. Characteristics of ICIs scRNA cohorts.

	**SKCM**[1]	**BCC**[2]
Patients		
Response	0	6
Non-response	11	4
Treatment naïve	13	0
Overall	24[1]	10[2]
Malignant cells	2142	1826
GEO Accession number	GSE115978	GSE123813

[1] Seven patients have been removed from GSE115978 due to lack of malignant cells data.
[2] One patient has been removed from GSE123813 due to its aberrant mutation and CNV profiles compared with other patients in this cohort.

Table S2. List of scRNA datasets applied to develop Stem.Sig.

Dataset Name	Cancer	Patients	Cells	Platform	PMID
GSE123813[2]	BCC	11	52884	10x Genomics	31359002
GSE143423[3]	BRCA	2	4375	10x Genomics	bioRxiv
SRP114962[4]	BRCA	8	2472	SNRS	29681456
GSE125449[5]	CHOL	10	5761	10x Genomics	31588021
GSE146771[6]	CRC	10	10468	Smart-seq2	32302573
GSE102130[7]	GBM	6	3321	Smart-seq2	29674595
GSE103224[8]	GBM	8	17185	Microwell	30041684
GSE131928a[9]	GBM	9	13553	10x Genomics	31327527
GSE131928b[9]	GBM	28	7930	Smart-seq2	31327527
GSE138794[10]	GBM	9	18458	10x Genomics	31554641
GSE139448[11]	GBM	3	12152	10x Genomics	32004492
GSE141982[12]	GBM	2	5263	10x Genomics	32105316
GSE148842[13]	GBM	7	111397	Microwell	bioRxiv
GSE70630[14]	GBM	6	4347	Smart-seq2	27806376
GSE84465[15]	GBM	4	3533	Smart-seq2	29091775
GSE89567[16]	GBM	10	6341	Smart-seq2	28360267
GSE103322[17]	HNSC	18	5902	Smart-seq2	29198524
GSE125449[5]	LIHC	9	3834	10x Genomics	31588021
GSE119926[18]	MB	25	7745	Smart-seq2	31341285
GSE117988[19]	MCC	1	10134	10x Genomics	30250229
GSE118056[19]	MCC	1	11024	10x Genomics	30250229
GSE117156[20]	MM	14	24918	MARS-seq	30523328
GSE140312[21]	NET	1	3158	10x Genomics	32054662
EMTAB6149[22]	NSCLC	8	40218	10x Genomics	29988129
GSE117570[23]	NSCLC	4	11453	10x Genomics	31033233
GSE127465[24]	NSCLC	7	31179	Smart-seq2	30979687
GSE143423[3]	NSCLC	3	12193	10x Genomics	bioRxiv
GSE118828[25]	OV	9	1909	Smart-seq2	30383866
CRA001160[26]	PAAD	35	57443	10x Genomics	31273297
GSE111672[27]	PAAD	3	6122	inDrop	31932730
GSE115978[1]	SKCM	31	7186	Smart-seq2	30388455
GSE72056[28]	SKCM	19	4645	Smart-seq2	27124452
GSE134520[29]	STAD	13	41554	10x Genomics	31067475
GSE139829[30]	UVM	11	103703	10x Genomics	31980621
Overall	17	345	663760		

Table S3. List of immunotherapy cohorts used in this study.

Cohorts	Cancer	Treatment (No. of patients)	All Patients	Response: R (CR/PR)	Response: NR (PD/SD)	Multi-variate cox regression analysis of overall survival: Events/Patients	TMB	Sex	Age	Purity
**Training and Validation**										
Braun 2020 RCC[31]	RCC	aPD-1	181	39	142	122/180	-	-	-	-
Mariathasan 2018 UC[32]	UC	aPD-L1	348	68	280	232/348	-	-	-	-
Liu 2019 SKCM[33]	SKCM	aPD-1	121	47	74	62/121	-	-	-	-
Gide 2019 SKCM[2][34]	SKCM	aPD-1 / Combo[3]	73	33	40	29/73	-	-	-	-
Riaz 2017 SKCM[35]	SKCM	aPD-1	49[4]	10	41	29/46	-	-	-	-
**Independent Testing**							-	-	-	-
Zhao 2019 GBM[36]	GBM	aPD-1	17[5]	10	7	9/17	-	√	√	-
Synder 2017 UC[37]	UC	aPD-L1	25	6	19	17/25	-	√	√	-
Hugo 2016 SKCM[38]	SKCM	aPD-1	26[5]	12	14	11/26	√	√	√	√
Van 2015 SKCM[39]	SKCM	aCTLA-4	36	14	22	24/36[6]	√	√	√	-
Kim 2018 GC[7][40]	GC	aPD-1	45	12	33	-	-	-	-	-

[1] During multi-variate cox regression analysis of overall survival, patients with missing covariates data have been deleted.

[2] Gide 2019 cohort had pre-ICT biopsies for melanoma patients treated with either anti-PD-1 (41 patients: 22 non-responders vs 19 responders) or the combination of anti-PD-1 and anti-CTLA-4 drugs (32 patients: 11 non-responders vs 21 responders).

[3] Combo: combined therapy of anti-PD-1 and anti-CTLA4.

[4] Two patients from Riaz 2017 were removed due to unknown response.

[5] Hugo 2016 comprises 27 pre-treated tumor samples from 26 patients. Zhao 2019 comprises 34 pre-treated tumor samples from 17 patients. We randomly selected a single tumor sample that represents the corresponding patient.

[6] Van 2015 has 2 patients deleted due to missingness during multi-variate cox regression analysis of overall survival.

[7] Kim 2018 does not include survival data.

Abbreviation: R: responder; NR: non-responder; RCC: renal cell carcinoma; UC: Urothelial carcinoma; SKCM: skin cutaneous melanoma; GBM: glioblastoma; aPD-1: anti-PD-1 antibody; aPD-L1: anti-PD-L1 antibody; TMB: tumor mutational burden; ITH: intratumor heterogeneity;

Table S4. List of CRISPR datasets.

CRISPR dataset	condition	cancer	Model cell
Freeman 2019[41]	NK[1]	Melanoma	B16
	OT1[2]	Melanoma	B16
Kearney 2018[42]	NK[1]	Colon	MC38
	OT1+IgG[3]	Colon	MC38
	OT1+PD1[4]	Colon	MC38
Manguso 2017[43]	GVAX[6]	Melanoma	B16
	GVAX+PD1[7]	Melanoma	B16
Pan 2018[44]	OT1[2]	Melanoma	B16
	Pmel-1_T[8]	Melanoma	B16
Patel 2017[45]	NY-ESO-1_MART-1 _T[9]	Melanoma	Mel624
Vredevoogd 2019[46]	MART1_T[10]	Melanoma	D10 IFNGR1$^-$
Lawson 2020[47]	Mid_CTL[5]	Breast	EMT6HA
	Mid_CTL[5]	Breast	X4T1HA
	Mid_CTL[5]	Colon	CT26HA
	Mid_CTL[5]	Colon	MC38OVA
	Mid_CTL[5]	Melanoma	B16OVA
	Mid_CTL[5]	Renal	RencaHA

[1] NK: natural killer cells;

[2] OT1: ovalbumin-specific transgenic CD8 T cells;

[3] OT1+IgG: OT1 + immunoglobulin;

[4] OT1+PD1: OT1 + anti–PD-1 antibody;

[5] Mid_CTL: cytotoxic cells at mid time point (defined as the population of cells following 1 or 2 treatment rounds);

[6] GVAX: a granulocyte-macrophage colony-stimulating factor (GM-CSF)-secreting, irradiated tumor cell vaccine;

[7] GVAX+PD1: GVAX + anti–PD-1 antibody;

[8] Pmel1_T: Pmel-1 T cells (specific for gp100 melanoma antigen);

[9] NY-ESO-1$^+$_MART-1$^+$ _T: CD8 T cells, which had been retrovirally transduced with a MART-1-specific and NY-ESO-1-specific T cell receptor (MART-1 T cells).

[10] MART-1_T: CD8 T cells, which had been retrovirally transduced with a MART-1-specific T cell receptor (MART-1 T cells).

Table S5. List of predictive gene expression signatures for immunotherapy

Signatures	Algorithms	Description
Stem.Sig	Naïve Bayes	Novel stemness signature identified by large-scale single cell RNA sequencing analysis
**Pan-cancer signatures**		
IFNG.Sig[48]	Average gene expression	An IFN-gamma-related profile that predicts PD-1blockade efficacy
T.cell.inflamed.Sig[48]	Average gene expression	T-cell inflamed gene expression profiles
PD-L1.Sig[49]	Expression of PD-L1	Gene expression profiles of PD-L1 / PDCD1.
LRRC15.CAF.Sig[50]	eigenWeightedMean*	Gene expression signature of LRRC15^{+} Cancer associated fibroblast
Cytotoxic.Sig[51]	Geometric mean of gene expression	Genes associated with cytotoxic activities
NLRP3.Sig[52]	ssGSEA	NLRP3 inflammasome-related genes derived from pan-cancer analysis
**Melanoma-specific signatures**		
IMPRES.Sig[53]	Comparison of 15 gene pairs	Twenty-eight immune checkpoints genes that build Immuno-predictive scores (IMPRES).
CRMA.Sig[54]	Geometric mean of gene expression	Genes of cancer-germline antigen that predicts the outcome of CTLA-4 blockade
TcellExc.Sig[1]	Overall Expression	Genes correlated with exclusion of T cells in the tumor microenvironment
IPRES.Sig[55]	Mean z-scores of GSVA scores	73 pathways involved with innate anti-PD-1 resistance (IPRES)
ImmuneCells.Sig[56]	Cancerclass	Gene expression signature of TREM2hi macrophages and γδ T cells
TRS.Sig[57]	GSVA	a tumor-reactive T cell signature
IMS.Sig[58]	Ratio of IMS scores / INF-γ scors[#]	Genes correlated with immunosuppression

*A method provided by R package multiGSEA *v1.1.99*

[#]IMS scores = Average gene expression of 18 immunosupression genes; IFN-γ scores = Average gene expression of 6 INF-γ genes

Table S6. Gene list of Stem.Sig.

ENO1	RBM17	RHCG	H1FX	UPF2	C19orf12	TXNDC5	SERPINA1	HNRNPH2	PHB2	LHX2	NNAT	CHCHD5	FAM204A	IFT20	COPA
UQCRH	KRT1	S100A7	RYK	RPP38	NUDT19	RNF11	VPS35	CEACAM6	PTMA	FOXG1	AC018890.6	CISD2	PCBP2	SMIM26	ACKR1
SERBP1	MYL6	GSDMC	HIST1H2AE	MSRB2	LRP3	EEF2	MTRNR2L1	C18ORF32	SELENOW	CTDNEP1	KCTD8	SPINK1	LECT1	MMP24OS	MADCAM1
BCL10	SLC25A3	LY6D	TPD52L1	EIF5AL1	LSM14A	SUMO3	SPHK1	TMED5	SLC25A6	ALKBH5	OOEP	RENBP	VRK1	ABCA7	FABP3
DENND2C	CNIH1	CALML5	PDCD2	MARVELD1	GPI	SHC4	HM13	DERL2	UBB	PIPOX	STEAP1B	MRPL23	CIB2	PVR	GPR137B
S100A6	SERF2	DSG1	NDUFA4	LDHB	GRAMD1A	RRS1	LBP	VPS29	YBX1	TRIM47	SLC26A3	NTS	PKMYT1	SELENOM	HTR2B
JTB	FEM1B	KRTDAP	NUPR2	PFDN5	SYNE4	SND1	MYDGF	ATP5G2	C11orf31	SMOX	MAMDC2	CLN3	RPA1	DESI1	ZNF330
LRRFIP2	ALDOA	CITED4	SHFM1	GTSF1	FAM98C	PRDX1	C19orf70	C9ORF78	ATP5B	C20orf24	PLEKHA7	PPP4C	ALYREF	PIGP	EIF4EBP3
TMA7	PFN1	ANKRD35	ATP5J2	PRSS27	DYRK1B	CFH	UBA52	POLR2E	MID1IP1	RNF126	SIK3	VCX2	SF3A2	UFD1L	SNX8
TMEM41A	PSMB3	NBPF14	C7orf73	TCEB2	EGLN2	C4BPA	TMPRSS3	ZFAND6	KNG1	SH3GL1	GGACT	ADAMTSL1	ASF1B	UBE2L3	RGS20
ATP5I	KRT10	MRPS21	ARHGEF35	BCL7C	AP2S1	AGT	RACK1	COMMD8	OMG	RAD23A	C14orf166	DKK2	DDA1	EIF5A2	MLANA
H2AFZ	H3F3B	C1orf43	FAM104B	IRX3	PIH1D1	KYNU	EIF3M	EMC3	SLC25A29	SSBP4	ALKBH7	EIF2S3	FUZ	PNCK	LRRN4CL
UBE2D2	ACTG1	APOA1BP	NGFRAP1	IRX5	UBE2S	ATP5G3	AGR2	C5ORF15	SNF8	KXD1	ZNF90	EMX2	PDXP	PTHLH	TM7SF3
NPM1	RIOK3	TAGLN2	VGLL1	CALB2	RBX1	UGT2B15	MTHFD2	RBM3	INAFM1	CCDC106	CDHR1	EPHA3	LMF2	AC007325.4	GNPTAB
TUBB	RALY	PIGC	SFRP1	KIF1C	DSCR8	GC	SUB1	SET	LINC00844	IGSF21	LMF1	EPHA7	NCAPH2	AL353997.6	ZNF106
PERP	TICAM1	SNRPE	ENY2	C1QBP	HNRNPA1	PPIA	C11ORF58	SUMO2	TXNL4A	PLPPR5	SHC3	NTN5	AC011043.1	MIR205HG	TSPAN10
ACTB	C19orf43	PPP2R5A	AARD	TMEM256	CHCHD2	IGFBP1	ERP29	DERL1	TMPRSS5	FAM89A	EHD2	WIF1	IGKC	NECTIN1	BIRC7
AC090498.1	TIAM1	H3F3A	SLURP1	EIF4A1	TSPYL5	PON3	TMED10	GRTP1	GLRX5	GRID2	POLE4	CENPU	SNORD100	ECM1	CLPTM1
PLP2	GRHL3	C1orf131	C8orf82	KRT19	AL365205.1	MTRNR2L10	RER1	C6ORF62	ATP5J	GNB2L1	POM121	SMC2	HOMER1	C1orf56	BAIAP2L2
EFNB1	PKP1	CEBPZOS	TPM2	ATP5G1	WDR77	DIAPH2	AP3S1	CASP6	ANKRD6	KLRC2	SERPINA3	GTSE1	CPEB4	SPRR1B	
SLC25A5	GPR87	COX7A2L	PRKCDBP	NME1	NDC1	FGL1	COPB1	BECN1	COX4I1	NKD1	STEAP3	PCP4	CDK13	SPRR2D	
PDP1	ADGRF4	PEX13	FTH1	HN1	FZD8	TXN	EEF1A1	PSMF1	PFKFB2	CYP27B1	GNB1	HPCA	TAC1	SFTPB	
CDKN2A	FAM83B	TCF7L1	EEF1G	FAM110A	TP53BP2	ORM1	FAU	ANP32B	CRYGS	C1ORF61	PARL	TMEM18	BEX5	FABP6	
CDKN2B	CALML3	RPIA	FAM89B	SNRPB	RSL1D1	ORM2	CCNDBP1	ATP5F1B	NUDT4	CMTM5	G6PD	POU4F3	TPH1	MUC21	
UBAP1	HCAR2	FAHD2A	CCS	COMMD7	EIF2S2	C5	AATF	HINT1	SPOCD1	ATP23	FAAP20	NRM	AGRN	SLC22A31	
SEC61B	ZNF750	DBI	DHCR7	BPI	DDOST	GAPDH	NOX1	HNRNPD	KCNF1	TTC9B	NR0B2	UBE2A	CGN	MRPL54	
ZBTB43	SERPINB2	NAA50	DGAT2	PSMA7	MKI67	TPI1	CHMP4A	LYPLA1	OSBPL6	C1ORF122	APH1A	MCTS1	SFTA2	NAPSA	
PPP1CA	TINCR	ROPN1	BARX2	MRGBP	AC012640.4	COX6A1	GSTA1	MTRNR2L2	MTHFD2L	C17ORF89	CRP	IDH3G	GCLC	AL031727.1	
GSTP1	IL36G	ROPN1B	ZMYND11	POP4	PRDX3	RAN	GTF3A	NACA	ARSJ	LINC01268	APOA2	TIMM23	FAM83A	QARS	

Table S7. Comparison of AUC of previous signatures in testing cohort.

**Pan-cancer signatures**	**Testing Cohort**	Hugo 2016 SKCM	Van 2015 SKCM	Kim 2018 GC	Zhao 2019 GBM	Synder 2017 UC
**Stem.Sig**	0.71	0.66	0.81	0.69	0.62	0.8
INFG.Sig	0.66	0.53	0.67	0.85	0.53	0.54
T.cell.inflamed.Sig	0.65	0.57	0.73	0.78	0.51	0.6
PDL1.Sig	0.62	0.52	0.69	0.79	0.46	0.51
LRRC15.CAF.Sig	0.6	0.6	0.52	0.55	0.63	0.81
NLRP3.Sig	0.6	0.46	0.67	0.62	0.74	0.46
Cytotoxic.Sig	0.44	0.43	0.29	0.77	0.57	0.49

**Melanoma-specific signatures**	**SKCM (Hugo2016 + Van 2015)**
IMPRES.Sig	0.81
CRMA.Sig	0.77
**Stem.Sig**	0.76
TcellExc.Sig	0.68
IPRES.Sig	0.66
ImmmunCells.Sig	0.62
TRS.Sig	0.57
IMS.Sig	0.53

Reference

1. Jerby-Arnon L, Shah P, Cuoco MS, Rodman C, Su M-J, Melms JC, et al. A Cancer Cell Program Promotes T Cell Exclusion and Resistance to Checkpoint Blockade. Cell. 2018;175:984-997.e24. https://doi.org/10.1016/j.cell.2018.09.006

2. Yost KE, Satpathy AT, Wells DK, Qi Y, Wang C, Kageyama R, et al. Clonal replacement of tumor-specific T cells following PD-1 blockade. Nat Med. 2019;25:1251–9. https://doi.org/10.1038/s41591-019-0522-3

3. Wang L, Dai J, Han R-R, Dong L, Feng D, Zhu G, et al. Single-Cell Map of Diverse Immune Phenotypes in the Metastatic Brain Tumor Microenvironment of Non Small Cell Lung Cancer. bioRxiv. 2019; https://doi.org/10.1101/2019.12.30.890517

4. Kim C, Gao R, Sei E, Brandt R, Hartman J, Hatschek T, et al. Chemoresistance Evolution in Triple-Negative Breast Cancer Delineated by Single-Cell Sequencing. Cell. 2018;173:879-893.e13. https://doi.org/10.1016/j.cell.2018.03.041

5. Ma L, Hernandez MO, Zhao Y, Mehta M, Tran B, Kelly M, et al. Tumor Cell Biodiversity Drives Microenvironmental Reprogramming in Liver Cancer. Cancer Cell. 2019;36:418-430.e6. https://doi.org/10.1016/j.ccell.2019.08.007

6. Zhang L, Li Z, Skrzypczynska KM, Fang Q, Zhang W, O'Brien SA, et al. Single-Cell Analyses Inform Mechanisms of Myeloid-Targeted Therapies in Colon Cancer. Cell. 2020;181:442-459.e29. https://doi.org/10.1016/j.cell.2020.03.048

7. Filbin MG, Tirosh I, Hovestadt V, Shaw ML, Escalante LE, Mathewson ND, et al. Developmental and oncogenic programs in H3K27M gliomas dissected by single-cell RNA-seq. Science. 2018;360:331–5. https://doi.org/10.1126/science.aao4750

8. Yuan J, Levitin HM, Frattini V, Bush EC, Boyett DM, Samanamud J, et al. Single-cell transcriptome analysis of lineage diversity in high-grade glioma. Genome Med. 2018;10:1–15. https://doi.org/10.1186/s13073-018-0567-9

9. Neftel C, Laffy J, Filbin MG, Hara T, Shore ME, Rahme GJ, et al. An Integrative Model of Cellular States, Plasticity, and Genetics for Glioblastoma. Cell. 2019;178:835-849.e21. https://doi.org/10.1016/j.cell.2019.06.024

10. Wang L, Babikir H, Müller S, Yagnik G, Shamardani K, Catalan F, et al. The phenotypes of proliferating glioblastoma cells reside on a single axis of variation. Cancer Discov. 2019;9:1708–19. https://doi.org/10.1158/2159-8290.CD-19-0329

11. Wang R, Sharma R, Shen X, Laughney AM, Funato K, Clark PJ, et al. Adult Human Glioblastomas Harbor Radial Glia-like Cells. Stem Cell Reports. 2020;14:338–50. https://doi.org/10.1016/j.stemcr.2020.01.007

12. Wang L, Catalan F, Shamardani K, Babikir H, Diaz A. Ensemble learning for classifying single-cell data and projection across reference atlases. Bioinformatics. 2020;36:3585–7. https://doi.org/10.1093/bioinformatics/btaa137

13. Zhao W, Dovas A, Spinazzi EF, Levitin HM, Banu MA, Upadhyayula P, et al. Deconvolution of cell type-specific drug responses in human tumor tissue with single-cell RNA-seq. Genome Med. 2021;13. https://doi.org/10.1186/s13073-021-00894-y

14. Tirosh I, Venteicher AS, Hebert C, Escalante LE, Patel AP, Yizhak K, et al. Single-cell RNA-seq

supports a developmental hierarchy in human oligodendroglioma. Nature. 2016;539:309–13. https://doi.org/10.1038/nature20123

15. Darmanis S, Sloan SA, Croote D, Mignardi M, Chernikova S, Samghababi P, et al. Single-Cell RNA-Seq Analysis of Infiltrating Neoplastic Cells at the Migrating Front of Human Glioblastoma. Cell Rep. 2017;21:1399–410. https://doi.org/10.1016/j.celrep.2017.10.030

16. Venteicher AS, Tirosh I, Hebert C, Yizhak K, Neftel C, Filbin MG, et al. Decoupling genetics, lineages, and microenvironment in IDH-mutant gliomas by single-cell RNA-seq. Science. 2017;355. https://doi.org/10.1126/science.aai8478

17. Puram S V., Tirosh I, Parikh AS, Patel AP, Yizhak K, Gillespie S, et al. Single-Cell Transcriptomic Analysis of Primary and Metastatic Tumor Ecosystems in Head and Neck Cancer. Cell. 2017;171:1611-1624.e24. https://doi.org/10.1016/j.cell.2017.10.044

18. Hovestadt V, Smith KS, Bihannic L, Filbin MG, Shaw MKL, Baumgartner A, et al. Resolving medulloblastoma cellular architecture by single-cell genomics. Nature. 2019;572:74–9. https://doi.org/10.1038/s41586-019-1434-6

19. Paulson KG, Voillet V, McAfee MS, Hunter DS, Wagener FD, Perdicchio M, et al. Acquired cancer resistance to combination immunotherapy from transcriptional loss of class I HLA. Nat Commun. 2018;9. https://doi.org/10.1038/s41467-018-06300-3

20. Ledergor G, Weiner A, Zada M, Wang SY, Cohen YC, Gatt ME, et al. Single cell dissection of plasma cell heterogeneity in symptomatic and asymptomatic myeloma [Internet]. Nat. Med. 2018. https://doi.org/10.1038/s41591-018-0269-2

21. Rao M, Oh K, Moffitt R, Thompson P, Li J, Liu J. Comparative scRNA-seq Reveals Liver Metastasis-Specific Targets in a Patient with Small Intestinal Neuroendocrine Cancer Running title : Comparative scRNA-seq of a Patient ' s Metastatic Cancer.

22. Lambrechts D, Wauters E, Boeckx B, Aibar S, Nittner D, Burton O, et al. Phenotype molding of stromal cells in the lung tumor microenvironment. Nat Med. 2018;24:1277–89. https://doi.org/10.1038/s41591-018-0096-5

23. Song Q, Hawkins GA, Wudel L, Chou PC, Forbes E, Pullikuth AK, et al. Dissecting intratumoral myeloid cell plasticity by single cell RNA-seq. Cancer Med. 2019;8:3072–85. https://doi.org/10.1002/cam4.2113

24. Zilionis R, Engblom C, Pfirschke C, Savova V, Zemmour D, Saatcioglu HD, et al. Single-Cell Transcriptomics of Human and Mouse Lung Cancers Reveals Conserved Myeloid Populations across Individuals and Species. Immunity. 2019;50:1317-1334.e10. https://doi.org/10.1016/j.immuni.2019.03.009

25. Shih AJ, Menzin A, Whyte J, Lovecchio J, Liew A, Khalili H, et al. Correction: Identification of grade and origin specific cell populations in serous epithelial ovarian cancer by single cell RNA-seq (PLoS ONE (2018) 13: 11 (e0206785) DOI: 10.1371/journal.pone.0206785). PLoS One. 2018;13:1–17. https://doi.org/10.1371/journal.pone.0208778

26. Peng J, Sun BF, Chen CY, Zhou JY, Chen YS, Chen H, et al. Single-cell RNA-seq highlights intra-tumoral heterogeneity and malignant progression in pancreatic ductal adenocarcinoma. Cell Res. 2019;29:725–38. https://doi.org/10.1038/s41422-019-0195-y

27. Moncada R, Barkley D, Wagner F, Chiodin M, Devlin JC, Baron M, et al. Integrating microarray-based spatial transcriptomics and single-cell RNA-seq reveals tissue architecture in

pancreatic ductal adenocarcinomas. Nat Biotechnol. 2020;38:333–42. https://doi.org/10.1038/s41587-019-0392-8

28. Tirosh I, Izar B, Prakadan SM, Wadsworth MH, Treacy D, Trombetta JJ, et al. Dissecting the multicellular ecosystem of metastatic melanoma by single-cell RNA-seq. Science. 2016;352:189–96. https://doi.org/10.1126/science.aad0501

29. Zhang P, Yang M, Zhang Y, Xiao S, Lai X, Tan A, et al. Dissecting the Single-Cell Transcriptome Network Underlying Gastric Premalignant Lesions and Early Gastric Cancer. Cell Rep. 2019;27:1934-1947.e5. https://doi.org/10.1016/j.celrep.2019.04.052

30. Durante MA, Rodriguez DA, Kurtenbach S, Kuznetsov JN, Sanchez MI, Decatur CL, et al. Single-cell analysis reveals new evolutionary complexity in uveal melanoma. Nat Commun. 2020;11. https://doi.org/10.1038/s41467-019-14256-1

31. Braun DA, Hou Y, Bakouny Z, Ficial M, Sant' Angelo M, Forman J, et al. Interplay of somatic alterations and immune infiltration modulates response to PD-1 blockade in advanced clear cell renal cell carcinoma. Nat Med. 2020;26:909–18. https://doi.org/10.1038/s41591-020-0839-y

32. Mariathasan S, Turley SJ, Nickles D, Castiglioni A, Yuen K, Wang Y, et al. TGFβ attenuates tumour response to PD-L1 blockade by contributing to exclusion of T cells. Nature. 2018;554:544–8. https://doi.org/10.1038/nature25501

33. Liu D, Schilling B, Liu D, Sucker A, Livingstone E, Jerby-amon L, et al. Integrative molecular and clinical modeling of clinical outcomes to PD1 blockade in patients with metastatic melanoma. Nat Med. 2019;25. https://doi.org/10.1038/s41591-019-0654-5

34. Gide TN, Quek C, Menzies AM, Tasker AT, Shang P, Holst J, et al. Distinct Immune Cell Populations Define Response to Anti-PD-1 Monotherapy and Anti-PD-1/Anti-CTLA-4 Combined Therapy. Cancer Cell. 2019;35:238-255.e6. https://doi.org/10.1016/j.ccell.2019.01.003

35. Riaz N, Havel JJ, Makarov V, Desrichard A, Urba WJ, Sims JS, et al. Tumor and Microenvironment Evolution during Immunotherapy with Nivolumab. Cell. 2017;171:934-949.e16. https://doi.org/10.1016/j.cell.2017.09.028

36. Zhao J, Chen AX, Gartrell RD, Silverman AM, Aparicio L, Chu T, et al. Immune and genomic correlates of response to anti-PD-1 immunotherapy in glioblastoma. Nat Med. 2019;25:462–9. https://doi.org/10.1038/s41591-019-0349-y

37. Snyder A, Nathanson T, Funt SA, Ahuja A, Buros Novik J, Hellmann MD, et al. Contribution of systemic and somatic factors to clinical response and resistance to PD-L1 blockade in urothelial cancer: An exploratory multi-omic analysis. Minna JD, editor. PLOS Med. 2017;14:e1002309. https://doi.org/10.1371/journal.pmed.1002309

38. Hugo W, Zaretsky JM, Sun L, Song C, Moreno BH, Hu-Lieskovan S, et al. Genomic and Transcriptomic Features of Response to Anti-PD-1 Therapy in Metastatic Melanoma. Cell. 2016;165:35–44. https://doi.org/10.1016/j.cell.2016.02.065

39. Van Allen EM, Miao D, Schilling B, Shukla SA, Blank C, Zimmer L, et al. Erratum for the Report "Genomic correlates of response to CTLA-4 blockade in metastatic melanoma" by E. M. Van Allen, D. Miao, B. Schilling, S. A. Shukla, C. Blank, L. Zimmer, A. Sucker, U. Hillen, M. H. Geukes Foppen, S. M. Goldinger, J. Utikal, J. C. Ha. Science. 2016;352:aaf8264–aaf8264. https://doi.org/10.1126/science.aaf8264

40. Kim ST, Cristescu R, Bass AJ, Kim K-M, Odegaard JI, Kim K, et al. Comprehensive molecular

characterization of clinical responses to PD-1 inhibition in metastatic gastric cancer. Nat Med. 2018;24:1449–58. https://doi.org/10.1038/s41591-018-0101-z

41. Freeman AJ, Vervoort SJ, Ramsbottom KM, Kelly MJ, Michie J, Pijpers L, et al. Natural Killer Cells Suppress T Cell-Associated Tumor Immune Evasion. Cell Rep. 2019;28:2784-2794.e5. https://doi.org/10.1016/j.celrep.2019.08.017

42. Kearney CJ, Vervoort SJ, Hogg SJ, Ramsbottom KM, Freeman AJ, Lalaoui N, et al. Tumor immune evasion arises through loss of TNF sensitivity. Sci Immunol. 2018;3:eaar3451. https://doi.org/10.1126/sciimmunol.aar3451

43. Manguso RT, Pope HW, Zimmer MD, Brown FD, Yates KB, Miller BC, et al. In vivo CRISPR screening identifies Ptpn2 as a cancer immunotherapy target. Nature. 2017;547:413–8. https://doi.org/10.1038/nature23270

44. Pan D, Kobayashi A, Jiang P, Ferrari de Andrade L, Tay RE, Luoma AM, et al. A major chromatin regulator determines resistance of tumor cells to T cell–mediated killing. Science. 2018;359:770–5. https://doi.org/10.1126/science.aao1710

45. Patel SJ, Sanjana NE, Kishton RJ, Eidizadeh A, Vodnala SK, Cam M, et al. Identification of essential genes for cancer immunotherapy. Nature. 2017;548:537–42. https://doi.org/10.1038/nature23477

46. Vredevoogd DW, Kuilman T, Ligtenberg MA, Boshuizen J, Stecker KE, de Bruijn B, et al. Augmenting Immunotherapy Impact by Lowering Tumor TNF Cytotoxicity Threshold. Cell. 2019;178:585-599.e15. https://doi.org/10.1016/j.cell.2019.06.014

47. Lawson KA, Sousa CM, Zhang X, Kim E, Akthar R, Caumanns JJ, et al. Functional genomic landscape of cancer-intrinsic evasion of killing by T cells. Nature. 2020;586:120–6. https://doi.org/10.1038/s41586-020-2746-2

48. Ayers M, Lunceford J, Nebozhyn M, Murphy E, Loboda A, Kaufman DR, et al. IFN-γ–related mRNA profile predicts clinical response to PD-1 blockade. J Clin Invest. 2017;127:2930–40. https://doi.org/10.1172/JCI91190

49. Topalian SL, Hodi FS, Brahmer JR, Gettinger SN, Smith DC, McDermott DF, et al. Safety, Activity, and Immune Correlates of Anti–PD-1 Antibody in Cancer. N Engl J Med. 2012;366:2443–54. https://doi.org/10.1056/nejmoa1200690

50. Dominguez CX, Müller S, Keerthivasan S, Koeppen H, Hung J, Gierke S, et al. Single-Cell RNA Sequencing Reveals Stromal Evolution into LRRC15 + Myofibroblasts as a Determinant of Patient Response to Cancer Immunotherapy. Cancer Discov. 2020;10:232–53. https://doi.org/10.1158/2159-8290.CD-19-0644

51. Rooney MS, Shukla SA, Wu CJ, Getz G, Hacohen N. Molecular and genetic properties of tumors associated with local immune cytolytic activity. Cell. 2015;160:48–61. https://doi.org/10.1016/j.cell.2014.12.033

52. Ju M, Bi J, Wei Q, Jiang L, Guan Q, Zhang M, et al. Pan-cancer analysis of NLRP3 inflammasome with potential implications in prognosis and immunotherapy in human cancer. Brief Bioinform. 2020;00:1–16. https://doi.org/10.1093/bib/bbaa345

53. Auslander N, Zhang G, Lee JS, Frederick DT, Miao B, Moll T, et al. Robust prediction of response to immune checkpoint blockade therapy in metastatic melanoma. Nat Med. 2018;24:1545–9. https://doi.org/10.1038/s41591-018-0157-9

54. Shukla SA, Bachireddy P, Schilling B, Galonska C, Zhan Q, Bango C, et al. Cancer-Germline Antigen Expression Discriminates Clinical Outcome to CTLA-4 Blockade. Cell. 2018;173:624-633.e8. https://doi.org/10.1016/j.cell.2018.03.026

55. Hugo W, Zaretsky JM, Sun L, Johnson DB, Ribas A, Lo RS, et al. Genomic and Transcriptomic Features of Response to Anti-PD-1 Therapy in Metastatic Melanoma Article Genomic and Transcriptomic Features of Response to Anti-PD-1 Therapy in Metastatic Melanoma. Cell. 2016;1–10. https://doi.org/10.1016/j.cell.2016.02.065

56. Xiong D, Wang Y, You M. A gene expression signature of TREM2hi macrophages and γδ T cells predicts immunotherapy response. Nat Commun. 2020;11:1–12. https://doi.org/10.1038/s41467-020-18546-x

57. Yan M, Hu J, Ping Y, Xu L, Liao G, Jiang Z, et al. Single-Cell Transcriptomic Analysis Reveals a Tumor-Reactive T Cell Signature Associated With Clinical Outcome and Immunotherapy Response In Melanoma. Front Immunol. 2021;12:1–14. https://doi.org/10.3389/fimmu.2021.758288

58. Cui C, Xu C, Yang W, Chi Z, Sheng X, Si L, et al. Ratio of the interferon-γ signature to the immunosuppression signature predicts anti-PD-1 therapy response in melanoma. npj Genomic Med. 2021;6:7. https://doi.org/10.1038/s41525-021-00169-w
