# Supplementary material for: Integrated analysis of single-cell and bulk RNA sequencing data reveals a pan-cancer stemness signature predicting immunotherapy response
Source: Genome Med. 2022 Apr 29;14:45. doi: 10.1186/s13073-022-01050-w (PMC9052621; doi:10.1186/s13073-022-01050-w)
Supplement: Supplementary file 2 — Additional file 2: Figure S1. Boxplots depicting the correlation of immune cells infiltration with Stem.Sig and TMB. Figure S2. Subgroup analysis of testing set. Figure S3. Correlation plot of Stem.Sig and other stemness signatures. Figure S4. Association Stem.Sig and expression level of TLS-related genes. [file 13073_2022_1050_MOESM2_ESM.pdf]

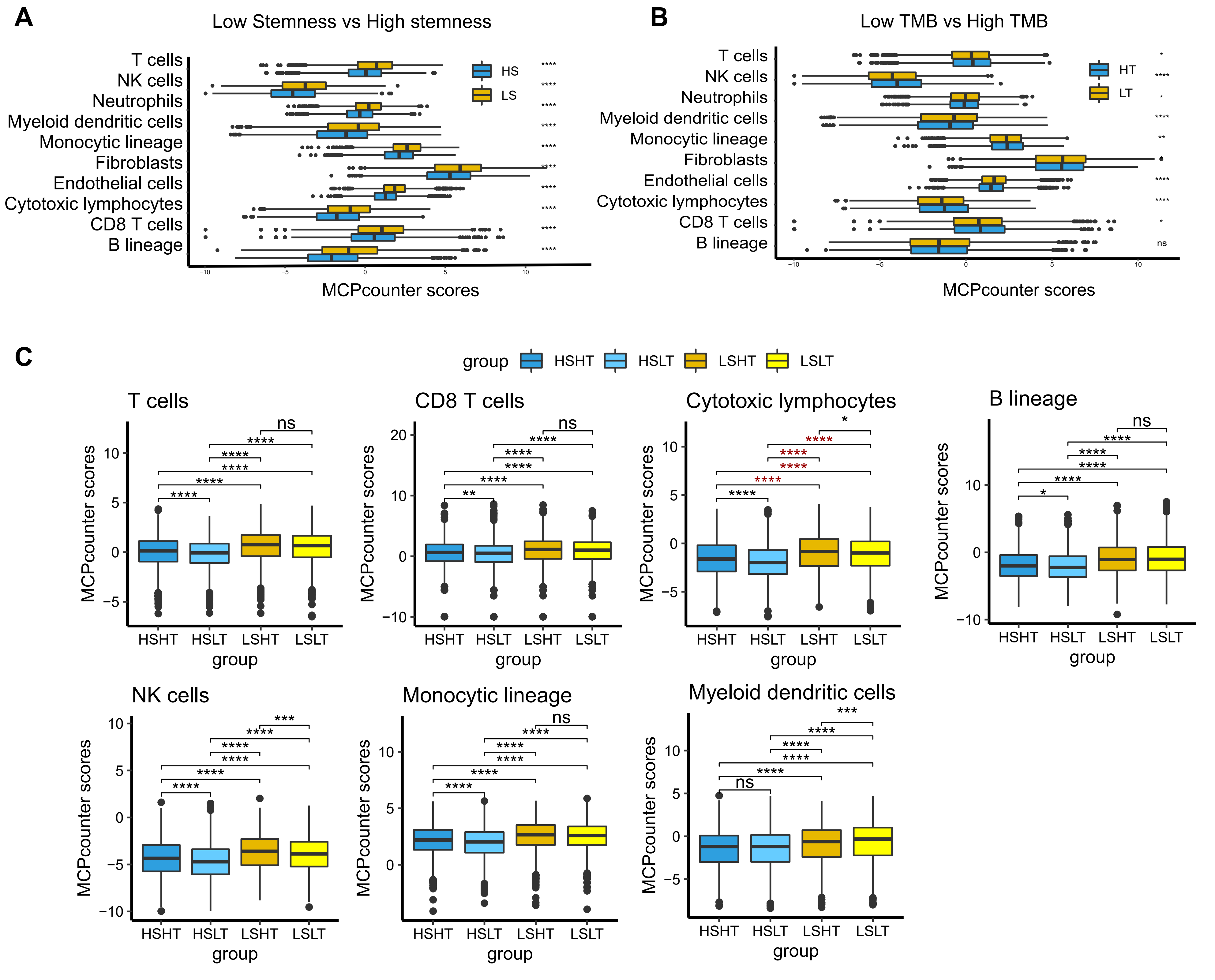

Figure S1. Boxplots depicting the correlation of immune cells infiltration with Stem.Sig and TMB. (Mann-Whitney U test; ns, not significant; *P < 0.05, **P < 0.01, ***P < 0.001, ****P < 0.0001)

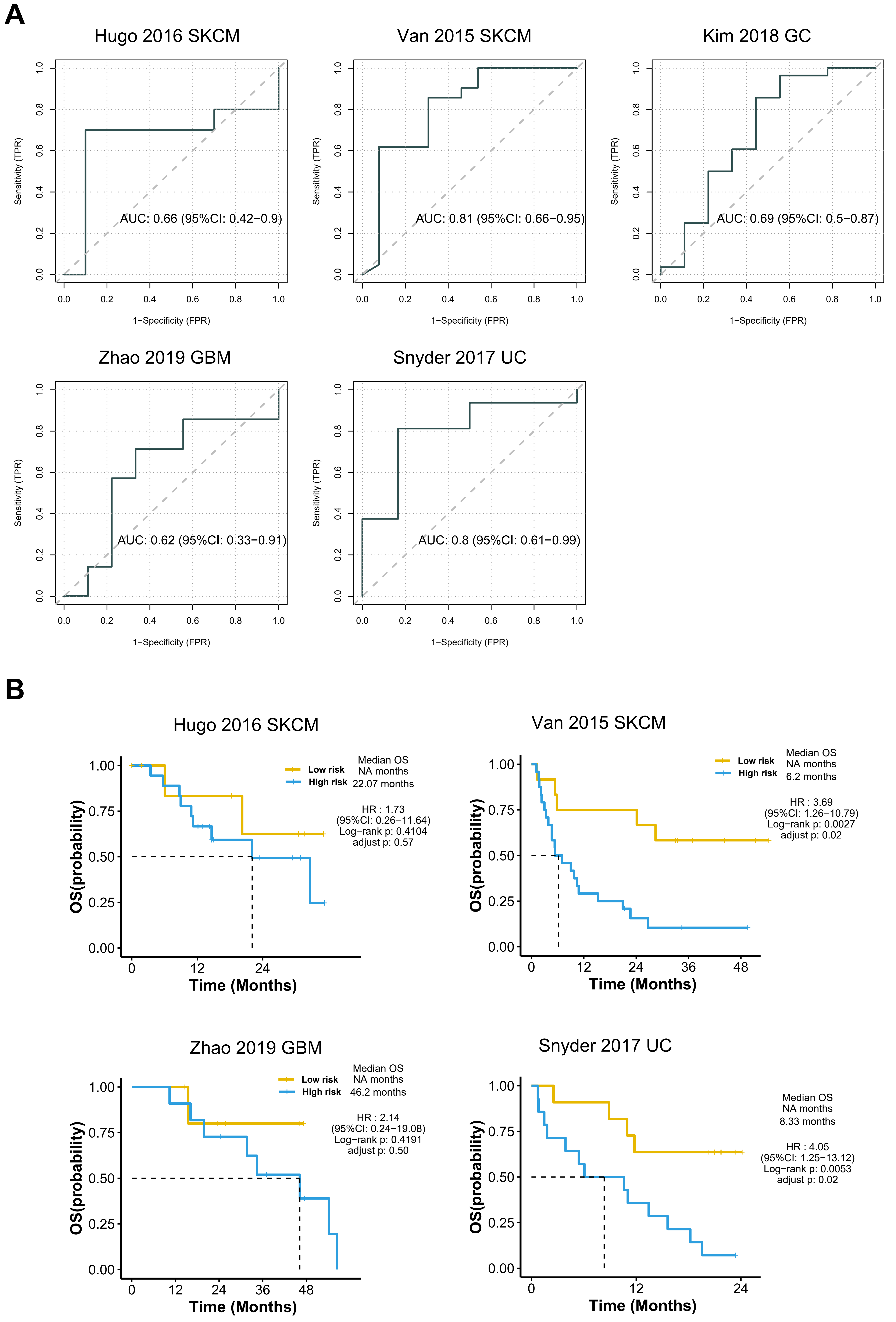

Figure S2. Subgroup analysis of testing set. A. ROC depicting the performance of Stem.Sig individual testing cohort. B. Kaplan-Meier survival plots of individual testing cohort.

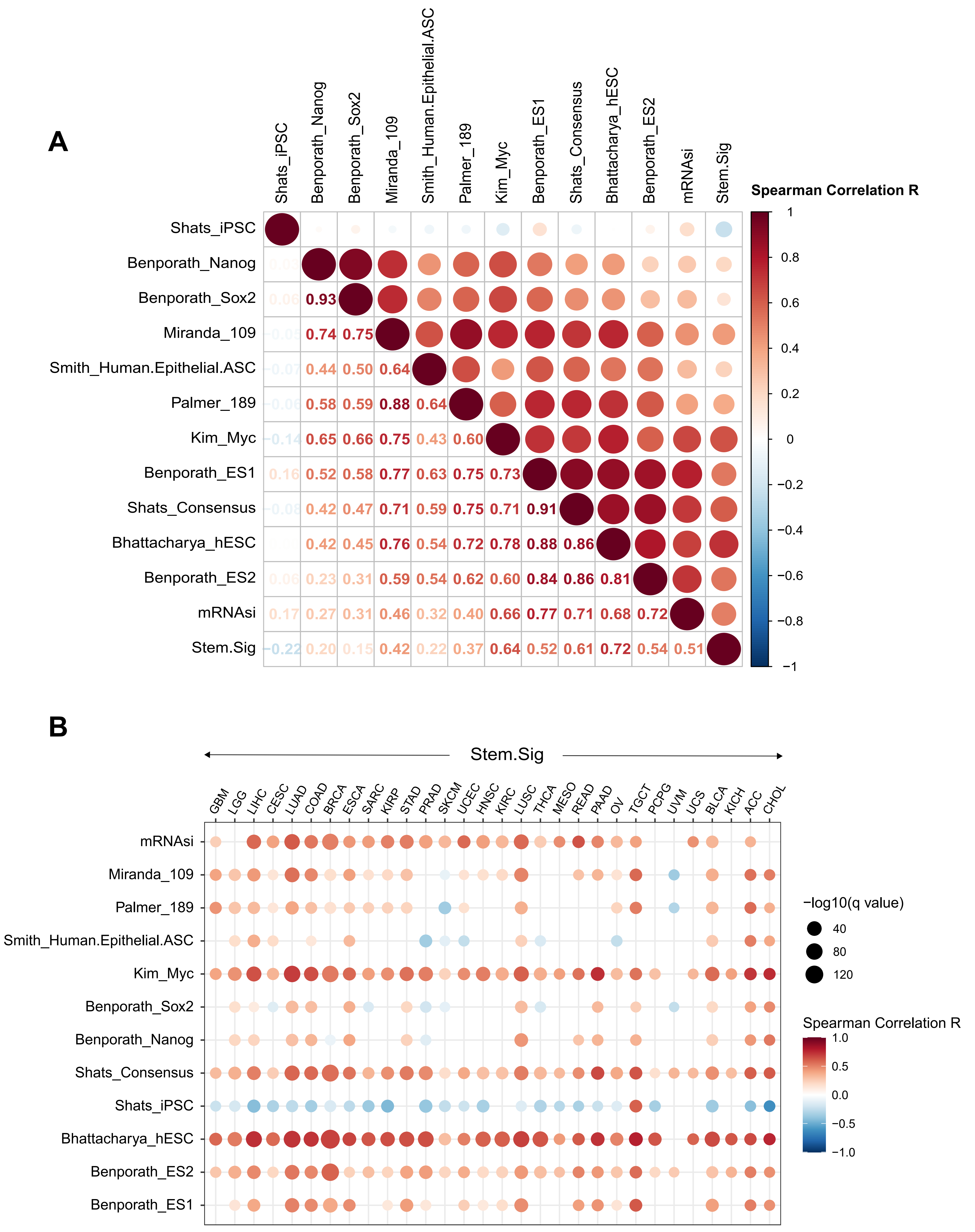

Figure S3. Correlation plot of Stem.Sig and other stemness signatures. Stem.Sig positively associated with all stemness signatures except Shats_iPSC, which does not show significantly positive correlation with any other stemness signature[1–6].

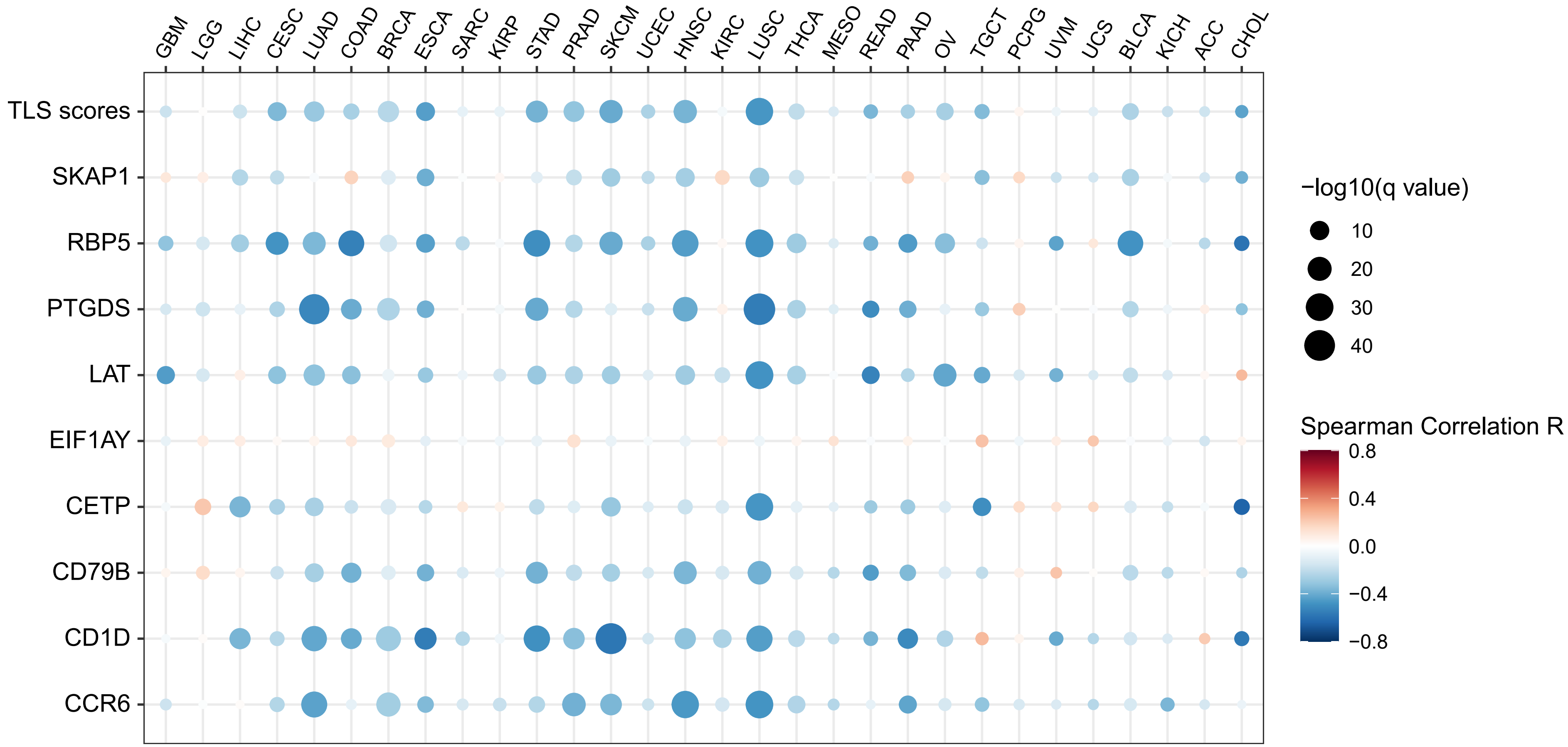

Figure S4. Association Stem.Sig and expression level of TLS-related genes. TLS score was calculated as the mean gene expression of TLS-related genes.

Reference

1. Malta TM, Sokolov A, Gentles AJ, Burzykowski T, Poisson L, Weinstein JN, et al. Machine Learning Identifies Stemness Features Associated with Oncogenic Dedifferentiation. Cell. 2018;173:338-354.e15. https://doi.org/10.1016/j.cell.2018.03.034
2. Miranda A, Hamilton PT, Zhang AW, Pattnaik S, Becht E, Mezheyeuski A, et al. Cancer stemness, intratumoral heterogeneity, and immune response across cancers. Proc Natl Acad Sci. 2019;116:9020–9. https://doi.org/10.1073/pnas.1818210116
3. Shats I, Gatza ML, Chang JT, Mori S, Wang J, Rich J, et al. Using a Stem Cell–Based Signature to Guide Therapeutic Selection in Cancer. Cancer Res. 2011;71:1772–80. https://doi.org/10.1158/0008-5472.CAN-10-1735
4. Ben-Porath I, Thomson MW, Carey VJ, Ge R, Bell GW, Regev A, et al. An embryonic stem cell–like gene expression signature in poorly differentiated aggressive human tumors. Nat Genet. 2008;40:499–507. https://doi.org/10.1038/ng.127
5. Palmer NP, Schmid PR, Berger B, Kohane IS. A gene expression profile of stem cell pluripotentiality and differentiation is conserved across diverse solid and hematopoietic cancers. Genome Biol. 2012;13:R71. https://doi.org/10.1186/gb-2012-13-8-r71
6. Bhattacharya B, Miura T, Brandenberger R, Mejido J, Luo Y, Yang AX, et al. Gene expression in human embryonic stem cell lines: unique molecular signature. Blood. 2004;103:2956–64. https://doi.org/10.1182/blood-2003-09-3314
